# Unraveling Water Sorption in Single‐Crystal MOFs: Insights from Spectroscopy and Modeling on the Role of Structure, Composition, and Guest Molecules

**DOI:** 10.1002/smll.73636

**Published:** 2026-05-06

**Authors:** Jonas Tittel, Fabian Knechtel, Orysia Zaremba, Andrea Darù, Jacopo Andreo, Laura Gagliardi, Stefan Wuttke, Evelyn Ploetz

**Affiliations:** ^1^ Department of Chemistry and Center for NanoScience (CeNS) Ludwig‐Maximilians‐Universität München Munich Germany; ^2^ BCMaterials Basque Center for Materials Leioa Spain; ^3^ Department of Chemistry Pritzker School of Molecular Engineering, and Chicago Center for Theoretical Chemistry University of Chicago Chicago Illinois USA; ^4^ Academic Centre for Materials and Nanotechnology AGH University of Krakow Krakow Poland

**Keywords:** DFT, NMR, MOFs, raman spectroscopy, single‐crystals, water harvesting

## Abstract

Understanding water sorption in metal‐organic frameworks (MOFs) is essential for advancing atmospheric water harvesting (AWH). Yet, most studies rely on bulk measurements that mask intrinsic material properties and particle heterogeneity. Here, we systematically examine how defect chemistry, residual guest molecules, and metal substitution shape water uptake in MOF‐801 and MOF‐808 at the single‐crystal level. Using bulk characterization together with in situ single‐crystal Raman spectroscopy, digestion ^1^H NMR, computational modeling, and long‐term cycling experiments, we disentangled how substituting Zr with Hf and changing topology affect the sorption behavior. Single‐crystal measurements reveal substantial crystal‐to‐crystal variations in residual DMF, which directly reduce the accessible pore volume and alter adsorption isotherms—effects hidden in ensemble‐averaged data. In both MOFs, Zr‐based materials exhibit higher uptake due to defect‐induced porosity. Hf substitution lowers the intrinsic defect density and improves cycling stability, but also leads to stronger solvent coordination and reduced pore accessibility. This trade‐off is most pronounced in MOF‐808(Zr), which shows high uptake but collapses during cycling, whereas the Hf analogue remains structurally stable at reduced capacity. By resolving how defects, guest molecules, and metal identity interplay at the single‐crystal level, this work provides molecular design rules for balancing stability and performance in next‐generation AWH sorbents.

## Graphical abstract

Water scarcity drives the search for efficient atmospheric water harvesting (AWH) materials. This study explores the impact of metal substitution (Zr to Hf) and linker exchange (fumaric to trimesic acid) on the water sorption performance of MOF‐801 and MOF‐808. Using Raman, NMR, and DFT simulations, we identify how Hf's higher electronegativity improves cycle stability and uptake efficiency, offering insights into optimizing MOFs for AWH.

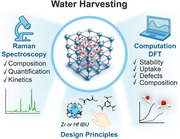

## 1 Introduction

Access to clean water is increasingly threatened by a growing population [1, 2], urbanization [3], and industry [4, 5] combined with climate extremes and deteriorating freshwater infrastructure [6, 7, 8, 9, 10, 11]. Atmospheric water harvesting (AWH), which extracts moisture directly from air across a broad range of relative humidities (RH), has emerged as a promising decentralized approach for generating potable water [12, 13, 14, 15]. Unlike fog collection or air‐cooling technologies that require specific climatic conditions, sorption‐based AWH systems can operate under mild thermal conditions and are suitable for both humid and arid climates [16, 17].

Among sorbent materials, metal–organic frameworks (MOFs) have attracted particular attention due to their exceptional structural tunability, sharp water‐adsorption steps, regeneration at low temperatures, and chemical robustness [18, 19, 20]. Their designable pore structures allow adsorption over nearly the entire RH range, making different MOFs suitable for ultradry deserts, tropical environments, cooling applications, or heat‐driven water generation [21, 22, 23, 24]. Zirconium‐ and aluminum‐cluster MOFs dominate current AWH research [24, 25, 26, 27, 28], while additional families continue to expand the accessible pore chemistries [25, 29].

Water uptake in these materials proceeds through well‐defined molecular steps determined by pore size: microporous MOFs (e.g., MOF‐801(Zr)), adsorb water initially at inorganic sites followed by hydrogen bonding, cluster growth, and pore filling [30], whereas mesoporous materials (e.g., MIL‐101(Cr) or MOF‐808) undergo capillary condensation and exhibit pronounced hysteresis [25, 31, 32]. Detailed spectroscopic and computational investigations have further shown that under nanoconfinement, hydrogen‐bonding networks reorganize in a highly cooperative manner and depend sensitively on local pore chemistry and adsorption sites [33]. Yet, the precise uptake pathways in individual MOF‐systems depend sensitively on topology, pore size, chemical composition, guest molecules, defects, crystal size, and morphology [20, 34, 35, 36, 37].

Most research has focused on optimizing MOF structures to improve pore capacity and water affinity, leading to an expanding library of new materials [32, 38]. Porosity effects on kinetics and uptake capacity have been extensively investigated [39], yet two major factors remain insufficiently understood: residual solvent molecules and defect chemistry. In fact, residual solvents such as N,N‐Dimethylformamid (DMF) or formic acid (FA) are known from both theory [40, 41] and experiment [42] to hinder, for instance, CO_2_ or CH_4_ adsorption. In some cases, even an enhanced selectivity of the CH_4_ adsorption is predicted [40], yet their quantitative influence on water adsorption has not been systematically evaluated. It is widely assumed that residual guest molecules suppress water uptake, yet no quantitative study has directly measured this effect at the level of the pristine MOF material. Defectivity adds further complexity: missing linkers and missing clusters alter porosity, create local adsorption sites, or trap water irreversibly [30, 43, 44], while their formation depends sensitively on modulators, synthesis conditions, metal identity, and crystal size.

A major obstacle is that nearly all available data arise from bulk characterization, which averages over thousands of crystals and cannot resolve particle‐to‐particle variations. Techniques such as N_2_ sorption, dynamic vapor sorption, powder X‐ray diffraction (PXRD), or thermogravimetric analysis (TGA) do not fully resolve the intrinsic heterogeneity of MOFs, like variations in morphology, defects, pore accessibility, and solvent retention, yet these differences at the nanoscale crucially determine uptake capacity, kinetics, and cycling durability.

In our previous work, we introduced a multimodal optical spectroscopy and in situ imaging correlation (MOSAIC) approach that integrates in situ Raman and nonlinear microscopy to enable single‐crystal (SC) analysis of MOFs under operational conditions [30, 45]. These studies revealed pronounced particle‐to‐particle heterogeneity in water adsorption, arising from differences in pore structure, local defects, and interparticle condensation pathways.

**FIGURE 1:**
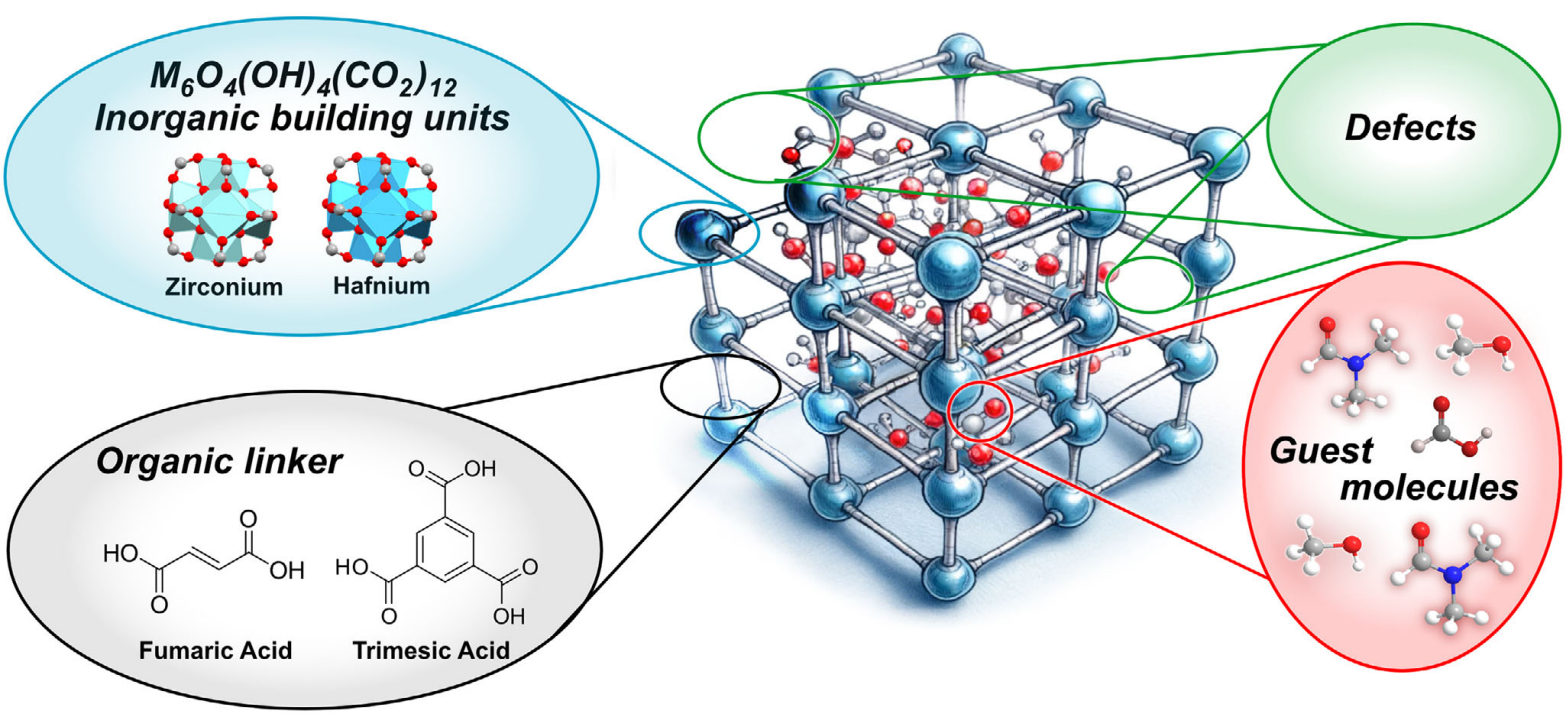
Key elements influencing water sorption in MOFs. Main factors impacting AWH in MOFs are metal cluster, linkers, defects, and residual guest molecules. To disentangle their contributions, we systematically varied the inorganic building unit by substituting Zr with Hf, exchanged fumaric acid with trimesic acid (chemical structure), resulting in the synthesis of MOF‐801 and MOF‐808, and investigated the impact of remaining guest molecules after synthesis.

Here, we extend this single‐crystal approach to dissect how metal identity (Zr vs. Hf), defect chemistry, pore architecture, and residual guest molecules collectively influence water uptake in two benchmark AWH MOFs: the microporous MOF‐801 and the mesoporous MOF‐808 (Figure 1). These materials share the same hexanuclear metal cluster but differ in linker, coordination number, and topology, allowing us to separate metal‐driven from structure‐driven effects. By substituting Zr with Hf, we probe how changes in metal‐ligand bond strength influence defect density, guest retention, pore accessibility, and ultimately water sorption kinetics and long‐term stability.

Using bulk characterization methods (scanning electron microscopy (SEM), XRD, sorption, proton nuclear magnetic resonance (^1^H NMR)), single‐crystal Raman microscopy and computational structural generation optimized at the density functional theory (DFT) level, we analyzed the chemical and structural composition of individual MOF crystals, quantified the contributions of missing linkers, missing clusters, guest molecules, and benchmarked their impact on water uptake capacity, kinetics and stability at the particle level. Single crystal Raman measurements revealed large crystal‐to‐crystal variations in residual DMF, which directly translates into substantial differences in water uptake even within a single batch. Computation confirmed that Hf–O bonds lead to fewer defects but stronger coordination of DMF and formate, stabilizing the framework yet reducing accessible pore volume. This trade‐off explains why Hfsubstituted MOF‐808 resists cycling‐induced collapse, whereas the Zr analogue fails, but also why Hf substitution lowers the maximum water uptake in both MOF‐801 and MOF‐808. Overall, this work provides a joint single‐crystal, spectroscopic, and computational investigation that disentangles how metal substitution, defect formation, and residual solvents jointly shape water sorption in Zr/Hf MOFs. These insights reveal the hidden heterogeneity of “identical” MOF crystals and establish design principles for rational engineering of sorbents with balanced pore accessibility, stability, and cycling performance for atmospheric water harvesting.

## 2 Results and Discussion

### 2.1 Synthesis

The synthesis of MOF‐801 and MOF‐808 (Zr and Hf) was carried out in DMF with identical starting material concentrations as well as synthesis, washing, and activation procedures to ensure a consistent comparison (Notes S1 and S2). SEM imaging revealed sharp octahedral morphologies, with strongly twinned single crystals of comparable sizes for each MOF type on the micrometer scale (Note S3.1). PXRD patterns were consistent with calculated diffraction data, showing no significant differences between Zr‐ and Hf‐based MOFs of the same structure (Note S3.2). N_2_ isotherms revealed that Zr‐based MOFs exhibit higher available surface areas than Hf‐based ones: 656 (Zr) vs. 341 m^2^ g^−^^1^ (Hf) for MOF‐801 and 1245 m^2^ g^−^^1^ (Zr) vs. 404 m^2^ g^−^^1^ (Hf) for MOF‐808, respectively, showing that the accessible volume in Hf‐based MOF scaffolds is reduced by more than half, despite identical crystal structure parameters.

### 2.2 Monitoring Water Adsorption in MOF‐801 Single‐Crystals

**FIGURE 2:**
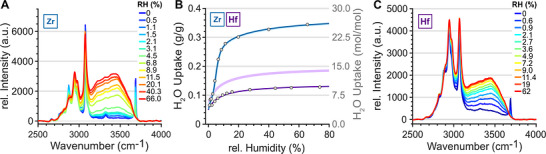
Quantification of water uptake in SC MOF‐801. (A,C) Raman spectra of MOF‐801(Zr) and MOF‐801(Hf) at varying humidities. (B) Water adsorption isotherms for single crystals of MOF‐801(Zr or Hf) corresponding to panels A and C, shown in g/gMof (left axis) and n/nMOF (transparent curve; right axis). Legend: MOF‐801/fumaric acid (squared icon). Data for Zr and Hf are displayed in blue and purple, respectively.

Water uptake was monitored by Raman spectroscopy in the spectral range of 3000–3700 cm^−^^1^. In this region, only signatures of the linker molecules are expected to contribute. However, for the dry MOF spectra, both aliphatic and aromatic C─H stretch vibrations (∼2850–2950 cm^−^^1^) were observed due to residual guest molecules, alongside the ν(═C─H) stretch mode of the linker molecules around 3070 cm^−^^1^ for all synthesized MOF scaffolds (Note S4). Upon exposure to vapor, the symmetric O─H stretch vibration of water centered between 3300–3600 cm^−1^ (Figure 2) was used to quantify the adsorbed water in the MOF scaffolds. Here, the area below the O─H stretch vibration was referenced to the characteristic ν(═C─H) stretch mode of the linker molecules (Note S1.4.3). Before recording SC isotherms, each MOF sample was additionally dried with a stream of dry nitrogen for 30 min on the microscope. Utilizing the spatial resolution of the Raman microscope, we monitored water uptake at the center of single crystals to probe the adsorption behavior of the pristine MOF material without interference from interparticle adsorption between neighboring particles (Note S3.4). We recorded its SC adsorption isotherm and quantified the total water uptake of the material. Water uptake was quantified in both g g_MOF_^−1^ and mol mol^−1^, as the g g_MOF_^−1^ ratio is biased by the heavier metal content in Hf‐based MOFs.

MOF‐801 exhibits an fcc crystal structure with *fcu*‐topology, constructed by linking hexanuclear metal clusters with twelve fumaric acid linkers. When monitoring the water uptake by Raman spectroscopy, MOF‐801(Zr) reveals a single‐crystal isotherm, classified as type V, which showed a multi‐step uptake process with a total uptake of about 0.37 g g_MOF_^−1^ in line with the literature (Figure 2A,B) [30, 35]. Strong water adsorption begins at approximately 10% relative humidity (RH), with water molecules initially binding to the inorganic building units (IBUs). The small offsets in the isotherms at 0% humidity originate from permanently bound water in the crystals that is not removed by the dry nitrogen stream. Importantly, our approach reflects realistic measurement conditions relevant for practical AWH applications, where it will not be possible to reactivate the MOFs at high temperature and high vacuum between every cycle. Adsorption is computationally described to progress through the smaller tetrahedral cages, where water has stronger adsorption energy (∼3 kcal mol^−1^), and subsequently through the octahedral cages (∼1 kcal mol^−1^).

In contrast, MOF‐801(Hf) exhibits a different adsorption behavior following a type I(b) isotherm with a total uptake of 0.14 g g_MOF_^−1^ (Figure 2B,C) [35]. Gradual exposure to humid air results in an immediate filling of micropores as the adsorbate concentration increases without a distinct plateau region. This suggests that MOF‐801(Hf) has a smaller accessible pore volume, pointing toward either changes in material density, defectivity or guest molecules, which we analyze in more detail in the following section.

### 2.3 Residual Guest Molecules and Defect Chemistry in MOF‐801

To identify the origin of the markedly different water sorption behaviors observed for MOF‐801(Zr) and MOF‐801(Hf), we first quantified the amount of residual guest molecules remaining in the frameworks after activation. Digestion ^1^H NMR (Note S5) was used to quantify DMF and FA remaining in the frameworks after activation. The spectra revealed significant differences in DMF content: while MOF‐801(Zr) retained only a small amount of DMF (linker‐to‐DMF ratio of 1:0.02), MOF‐801(Hf) incorporated roughly ten times more DMF. Raman spectra corroborate this trend through the intensity of aliphatic C─H stretching bands at 2850–2950 cm^−1^ (Figure 2A,C; Note S4). Because DMF interacts strongly with the μ_3_─OH groups of the metal clusters and occupies pore space, these differences in solvent retention are expected to strongly influence accessible pore volume and water sorption behavior.

**FIGURE 3:**
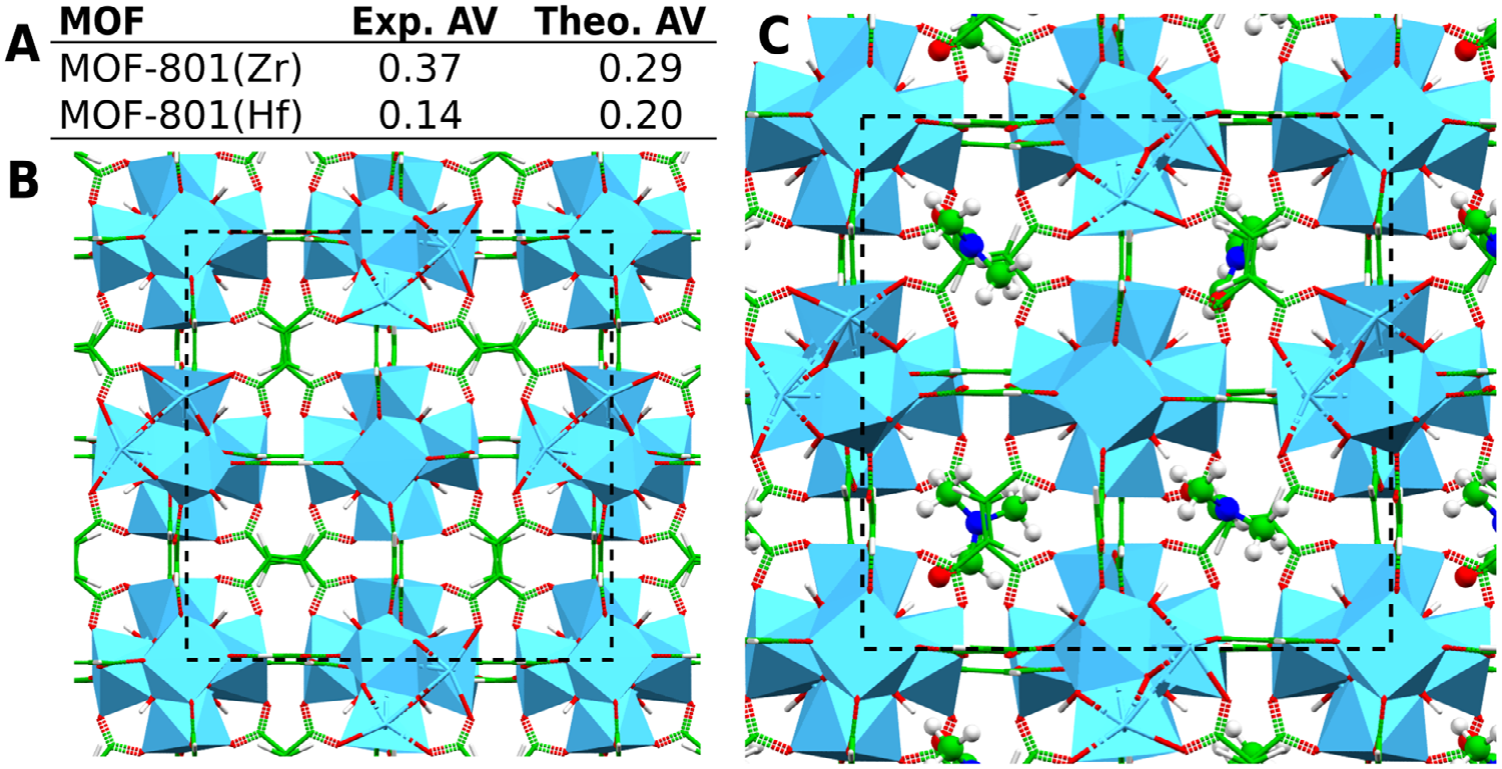
Uptake capacities of MOF‐801. (A) Available pore volume (g gMOF−1) for water uptake obtained from SC isotherms via Raman spectroscopy and DFT calculations. (B, C) Structural models of MOF‐801: (B) ideal framework and (C) structure containing four DMF molecules per unit cell in place of linkers (shown in ball‐and‐stick representation). Color code: H (white), O (red), C (green), Zr/Hf (cyan).

To disentangle the influence of intrinsic defects and guest molecules in shaping pore accessibility for atmospheric water harvesting, we performed DFT calculations on computationally generated structural models of on MOF‐801(Zr) and MOF‐801(Hf) in both pristine and defective forms (Figure 3; Note S6). For pristine MOF‐801(Zr), the theoretically available pore volume of 0.29 g g_MOF_^−1^ was approximately 28% lower than the experimental value of 0.37 g g_MOF_^−1^ (Figure 2B). This discrepancy suggests that defects contribute to the enhanced experimental uptake in MOF‐801(Zr). Missing‐linker defects alone cannot fully account for the increased capacity, whereas a single missing‐cluster would – in the absence of guest molecules – lead to an available pore volume exceeding the experimental value (Note S6.1). The most plausible explanation for the observed uptake behavior in MOF‐801(Zr) is therefore a combination of missing linkers, missing clusters, and partial pore blocking by DMF molecules. In contrast, pristine MOF‐801(Hf) is predicted to have a theoretical available pore volume of 0.20 g g_MOF_^−1^, exceeding the experimental value of 0.14 g g_MOF_^−1^ (Figure 3).

For MOF‐801(Hf), the dominant factor limiting pore accessibility is the presence of residual DMF molecules occluding pore space or coordinating to μ_3_─OH groups. Additional DFT simulations confirm a continuous decrease in available pore volume as the number of incorporated DMF molecules inside the Hf‐based framework increases (Figure 3; Table S8). The experimental accessible pore volume of MOF‐801(Hf) can be reproduced by structures containing either (i) three missing linkers with six DMF molecules per unit cell, or (ii) four missing linkers with eight DMF molecules per unit cell, both yielding an available pore volume or approximately 0.148 g g_MOF_^−1^. The measured value of 0.138 g g_MOF_^−1^ likely reflects an ensemble of unit cells with different combinations of defects and guest molecules.

Taken together, these results suggest that: (i) in MOF‐801(Zr), defects caused by missing linkers and clusters increase pore accessibility beyond what would be expected from their ideal structure despite partial pore blocking by residual DMF; (ii) in MOF‐801(Hf), stronger DMF coordination together with a lower intrinsic defect density lead to a decrease in accessible pore volume compared to the pristine model; and (iii) different levels of DMF in the Zr‐ and Hf‐based frameworks critically determine the amount of water taken up.

### 2.4 Systematic Variation of Residual DMF in MOF‐801

Both experiments and simulations indicated that the pore volume available for AWH in MOF‐801 strongly depends on the amount of residual DMF confined within the framework. To examine this effect more closely, we prepared a series of seven MOF‐801(Zr) samples with systematically varied DMF contents. All batches were synthesized under identical conditions. Differences appeared only because of post‐synthetic washing procedures, which went from no washing to thorough, multi‐step solvent exchange (Note S7). After washing, each batch was activated for 24 h to ensure an identical thermal treatment. Digestion ^1^H NMR confirmed a wide spread in DMF‐to‐linker ratios: the unwashed material contained nominally 0.647 DMF molecules per fumarate linker, whereas the most thoroughly washed sample showed only 0.002 DMF molecules per linker (Note S7.2.2). Single‐crystal Raman spectroscopy further showed that the DMF content varied not only between batches but also between individual crystals in the same batch. This variation was particularly large for minimally washed samples, where ratios between 0.42 and 0.82 DMF molecules per linker were detected (Note S7.3). This pronounced particle‐to‐particle heterogeneity highlights that bulk characterization alone does not fully capture the distribution of guest molecules in MOF‐801 and emphasizes the importance of single‐crystal measurements.

**FIGURE 4:**
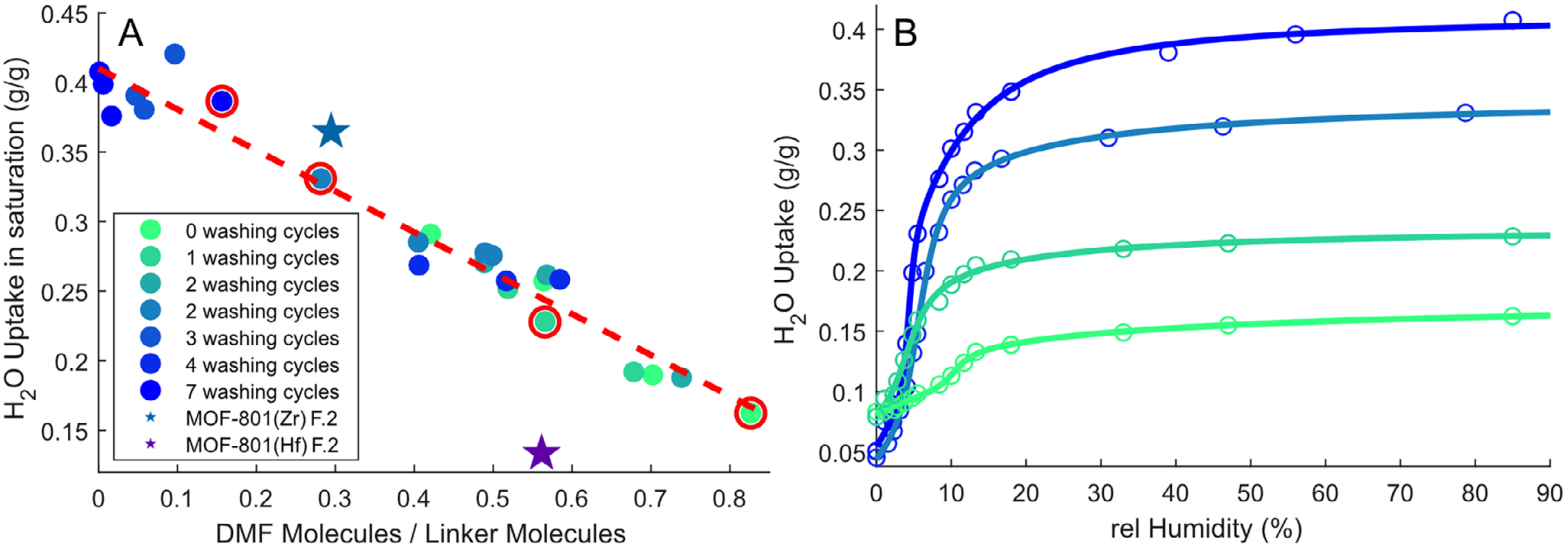
Correlation between residual DMF content and water uptake in MOF‐801(Zr). (A) Saturated water uptake of three single MOF‐801(Zr) crystals per washing condition plotted as a function of their DMF‐to‐linker ratios. Colors distinguish the seven synthesis/washing batches. The red dashed line represents a linear fit to all data (*y* = −0.29x +0.41). Star symbols indicate the reference isotherms for MOF‐801(Zr) (blue) and MOF‐801(Hf) (purple) shown in Figure 2B. (B) Full single‐crystal water adsorption isotherms of the representative crystals marked by red circles in panel (A).

After determining the DMF content of each individual crystal, three single crystals per batch were subjected to water adsorption measurements to obtain the corresponding single‐crystal isotherms. Figure 4A plots the saturated water uptake of each crystal against its independently measured DMF‐to‐linker ratio. Although crystals originating from the same batch (same color) show substantial scatter along both axes – reflecting the intrinsic particle‐to‐particle heterogeneity – each data point falls onto a common linear trend once treated individually. This correlation demonstrates that residual DMF systematically suppresses water uptake in MOF‐801(Zr), and that high DMF contents can reduce the accessible pore volume by more than 50%. The MOF‐801(Zr) isotherm shown previously in Figure 2 aligns nicely with the trend.

As seen in Figure 4A, the reference point and uptake ability of MOF‐801(Hf) lie substantially below the DMF‐uptake trend established for MOF‐801(Zr). The reduced water uptake of the Hf‐based framework can therefore not be attributed to its higher DMF content alone. Instead, the lower performance results from a combination of factors: slightly smaller intrinsic pore size, fewer defects because of stronger Hf–fumarate bonds, and more strongly bound residual DMF that limits pore accessibility. These observations are in good agreement with DFT‐predicted available volumes of 0.29 g g_MOF_^−1^ for pristine MOF‐801(Zr) and 0.20 g g_MOF_^−1^ for pristine MOF‐801(Hf).

Figure 4B presents the single‐crystal isotherms for the representative data points in Figure 4A. Crystals with low DMF content exhibit a type‐V isotherm with steep uptake at moderate relative humidities, as discussed earlier for MOF‐801(Zr). However, crystals containing a high amount of DMF show only minimal adsorption at low RH and a delayed uptake at higher RH, consistent with a transition toward type‐IV behavior. These high‐DMF crystals also retain appreciable amounts of water at RH = 0%, indicating that coordinated or pore‐confined water cannot be removed by nitrogen flow alone. Such water retention poses an additional limitation for atmospheric water harvesting, as it both reduces the effective desorption efficiency and further diminishes the accessible pore volume.

### 2.5 Monitoring Water Adsorption in MOF‐808 Single‐Crystals

To assess how pore size and framework topology influence water absorption in Zr‐ and Hf‐based MOFs, we extended our single‐crystal Raman analysis from the microporous MOF‐801 to its mesoporous analogue, MOF‐808 (Figure 1). MOF‐808 adopts an *spn*‐topology. It is composed of hexanuclear metal clusters coordinated by six trimesic acid linkers and six formate modulators, generating a system of tetrahedral and adamantane‐like cages. The larger pore structure of MOF‐808 results in a total pore volume about twice as large as that of MOF‐801, in agreement with N_2_ sorption experiments.

**FIGURE 5:**
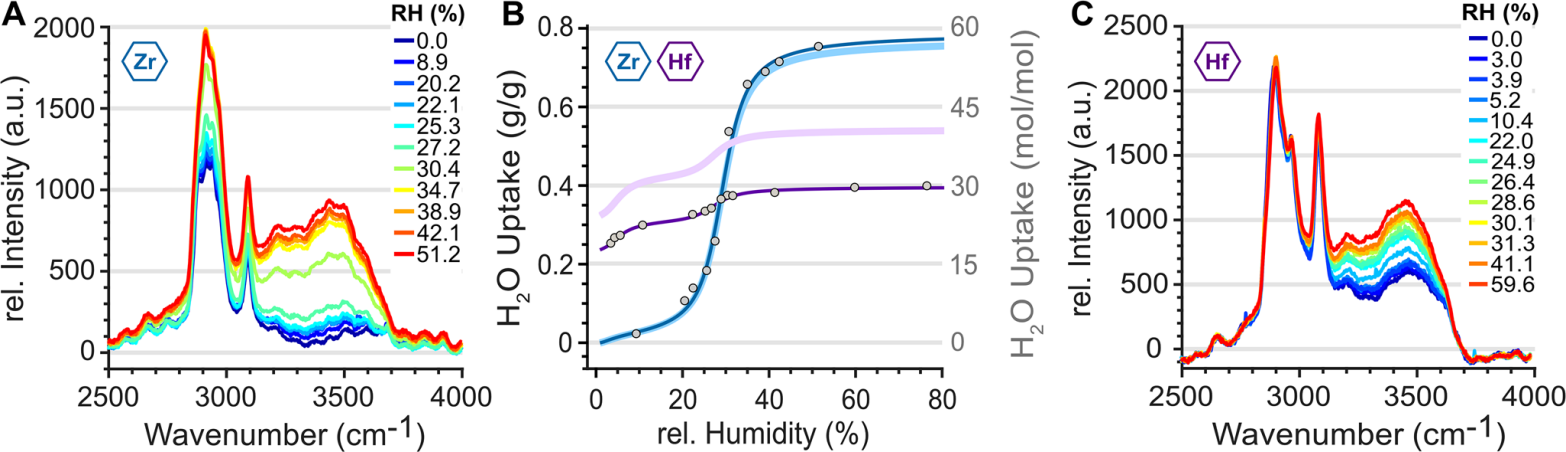
Quantification of water uptake in SC MOF‐808. (A,C) Raman spectra of MOF‐808(Zr) and MOF‐808(Hf) recorded at increasing humidities. (B) Corresponding SC water adsorption isotherms for Zr‐ and Hf‐ based MOF‐808, shown in g/gMof (left axis) and n/nMOF (transparent curve; right axis). Legend: MOF‐808/trimesic acid (hexagonal icon). Data for Zr and Hf are displayed in blue and purple, respectively.

Raman spectra of dry MOF‐808 contain the typical ν(C─H) stretching vibration of trimesic acid at 3069 cm^−^^1^, as well as the μ_3_─OH stretching mode at 3682 cm^−^^1^, which suggests seed water is already coordinated to the inorganic building unit (Figure 5A; Note S4). In MOF‐808(Zr), only very small amounts of free water are detected between 3300–3600 cm^−^^1^ for relative humidities below ∼25% (Figure 5A; blue graphs). Upon exposure to increasing humidity, however, the material shows a steep increase in OH‐stretch intensity between 26% and 38% RH, corresponding to a total uptake of 0.78 g g_MOF_^−1^. The resulting single‐crystal isotherm displays a type IV isotherm, starting with a small shoulder at low humidity followed by a sharp, cooperative adsorption step at intermediate RH (Figure 5B). This uptake behavior can be explained by the sequential filling of microporous tetrahedral cavities, followed by the larger adamantane‐like mesopores [43].

MOF‐808(Hf) absorbs about half the water of its Zr analogue with a maximum loading of 0.40 g g_MOF_^−1^. This lower capacity is consistent with a decreased Brunauer–Emmett–Teller (BET) surface area observed for MOF‐808(Hf), suggesting a smaller effectively available pore volume. Water adsorption starts earlier (∼ 25% RH) than in MOF‐808(Zr), which indicates a higher affinity of the Hf‐based framework for water. This stronger affinity, however, is accompanied by significant water retention. Even prolonged drying under a nitrogen stream does not fully remove the adsorbed water at low RH. MOF‐801(Hf) retains about 0.23 g g_MOF_^−1^ of water, corresponding to about 25% of its total uptake. The presence of strongly bound (or trapped) water points to partial pore blockage and limited desorption efficiency, consistent with the DMF‐related pore obstruction observed in MOF‐801(Hf). Together, these factors reduce both the overall accessible pore volume and desorption efficiency, which in turn limits the suitability of MOF‐808(Hf) for atmospheric water‐harvesting applications.

### 2.6 Residual Guest Molecules and Defect Chemistry in MOF‐808

To understand the origin of the reduced uptake and increased water retention observed for MOF‐808(Hf), we next examined the defect chemistry and residual guest content of both MOF‐808 variants. Digestion ^1^H NMR spectroscopy revealed comparable formate‐to‐linker ratios for MOF‐808(Zr) and MOF‐808(Hf), indicating similar levels of modulator incorporation. In contrast, the DMF content of the Hf‐based analogue was moderately but consistently higherapproximately a 20% increase relative to the Zr framework (Note S5). Although this difference is less pronounced than in MOF‐801, it nonetheless suggests that tighter Hf–linker binding may hinder the removal of coordinated or pore‐confined DMF during activation.

^1^H NMR further revealed a higher fraction of trimesic acid per linker molecule in MOF‐808(Hf), implying partial over‐coordination at equatorial sites of the Hf_6_ nodes. Such additional trimesic acid linkers effectively constrict the pore apertures leading into the adamantane cages. Consequently, water molecules entering these cavities desorb only incompletely—consistent with the substantial quantity of water retained at RH = 0% in the single‐crystal isotherm. Thus, both the increased linker content and the higher DMF content in MOF‐808(Hf) likely contribute to obstructed diffusion and reduced desorption efficiency.

**FIGURE 6:**
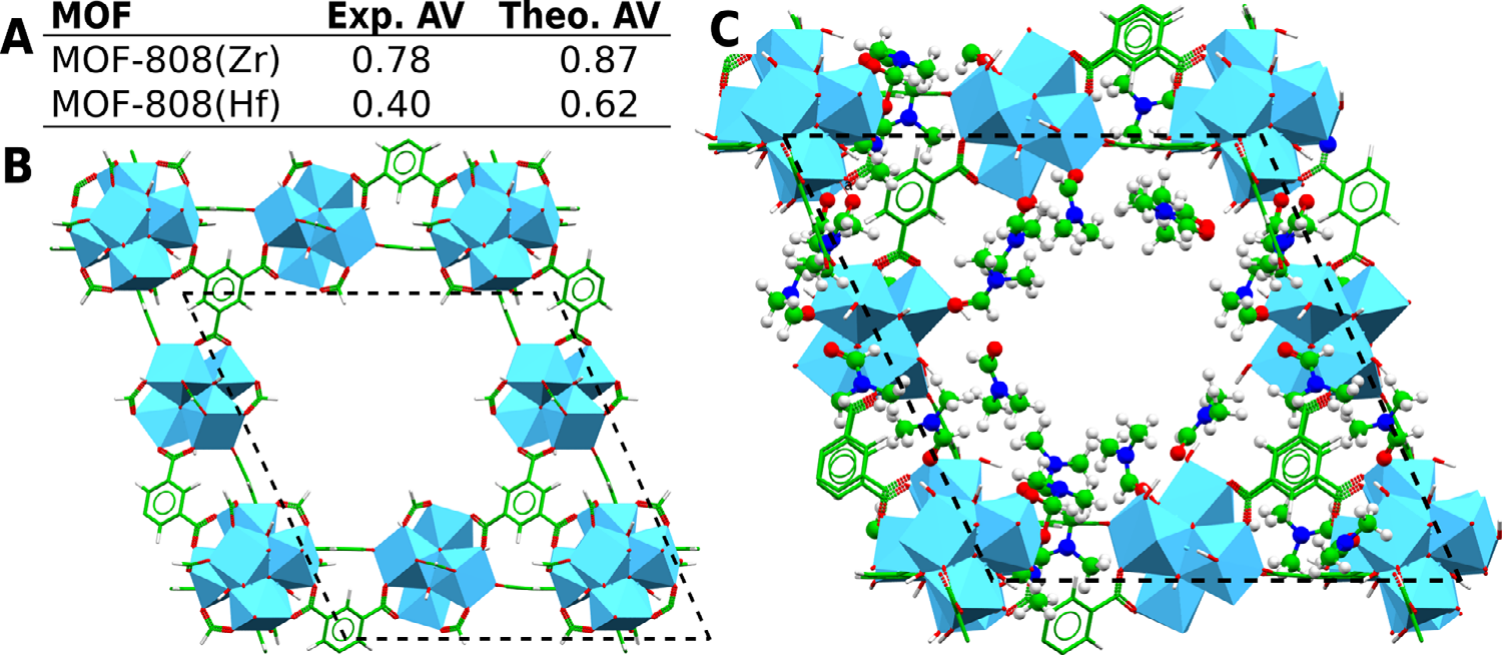
Uptake capacities of MOF‐808. (A) Available volume (g gMOF^−1^) for water uptake obtained from SC isotherms via Raman spectroscopy and DFT calculations. (B, C) Framework structures of MOF‐808 shown with different types of defects: (B) pristine network, (C) missing linkers and added DMF molecules. Elements are colored: hydrogen atoms (white), oxygen atoms (red), carbon atoms (green), and zirconium or hafnium (cyan).

Comparison between experimental and theoretical available volumes provides additional mechanistic insight. For MOF‐808(Zr), the defect‐free calculated available pore volume of simulated MOF‐808(Zr) of 0.87 g g_MOF_^−1^ exceeds the experimental value of 0.78 g g_MOF_^−1^ by 11% (Figure 6). This discrepancy is consistent with a small number of DMF molecules occupying the tetrahedral cages, where computation shows that DMF binds more strongly to the µ_3_─OH groups than water (Note S6). Such binding explains the shoulder observed at low RH in the Zr‐based SC isotherm and the complete absence of retained water at 0% RH: DMF displaces water efficiently but does not trap it.

In contrast, MOF‐808(Hf) exhibits a much larger discrepancy between theory (0.62 g g_MOF_^−1^) and experiment (0.40 g g_MOF_^−1^), corresponding to a 36% reduction. DFT simulations confirm that missing‐linker defects alone cannot account for this loss in pore volume, as such defects are energetically disfavored in Hf frameworks due to stronger Hf─O bonding (Note S6). Instead, the experimental accessible pore volume can be reproduced only when DMF is explicitly included in the pores: the presence of just one or two pore‐confined DMF molecules per unit cell already reduces the theoretical capacity to 0.39 g g_MOF_^−1^ in DMF‐containing MOF‐808(Hf) models. The results showed that the Hf‐based material possesses fewer intrinsic defects but retains strongly bound DMF molecules that persist even after activation and substantially reduce its effective porosity.

Despite incorporating similar amounts of DMF after activation, MOF‐808(Zr) and MOF‐808(Hf) display markedly different water sorption behavior. MOF‐808(Zr) releases all water at 0% RH, whereas MOF‐808(Hf) retains 42% of its total capacity. These differences arise not only from stronger Hf–linker interactions, which suppress defect formation, but also from stronger Hf–DMF binding (Note S6), which increases pore blocking and impedes desorption. As in MOF‐801, the Hf‐based analogue thus combines reduced defectivity with stronger solvent coordination, leading to increased structural stability but at the cost of significantly diminished water‐harvesting performance due to persistent solvent coordination and hindered desorption.

Computational simulations further rationalize this stabilization of MOF‐808 upon Hf substitution. The key factor is that Hf‐based frameworks are likely to form fewer defects than their Zr counterparts. This stabilization originates from the higher electron affinity of Hf relative to Zr, [46] which strengthens the Hf─O bonds within the node‐linker coordination sphere. The enhanced electron‐accepting character of Hf^4^^+^ increases its attraction to electron‐rich ligands—including DMF—thereby reinforcing node–linker interactions and further suppressing defect formation. These stronger interactions also promote the retention of DMF within the pore network, preventing water molecules from acting as seed molecules for hydrogen‐bonding chains and ultimately hindering both water uptake and desorption. Thus, while hafnium substitution enhances the structural robustness of MOF‐808, it simultaneously exacerbates solvent blocking and reduces the operational water‐harvesting efficiency of the framework.

### 2.7 Long‐Term Water Cycling of MOF Single Crystals

In addition to high water adsorption capacity, fast adsorption/desorption kinetics and long‐term stability across multiple adsorption/desorption cycles are critical for AWH applications. Because Hf‐based MOFs exhibit fewer linker‐related defects, we evaluated the dynamic stability of all four MOF materials by performing rapid sorption cycling experiments. Individual single crystals were alternately exposed to dry (0%) and moderately humid (∼40% RH) nitrogen streams in 60 s intervals, while the integrated O─H stretch intensity was recorded via Raman spectroscopy with a temporal resolution of 1 s for MOF‐801 and 2 s for MOF‐808. All materials reached adsorption equilibrium within ∼5 s (Figure S27), consistent with the short diffusion paths in micron‐sized single crystals. In contrast, desorption kinetics depended strongly on pore size and solvent occupancy: MOF‐801 required ∼30 s for complete water release due to nanoconfined water in DMF‐occupied pores [47], whereas MOF‐808 showed nearly instantaneous desorption, reflecting its larger accessible cavities.

**FIGURE 7:**
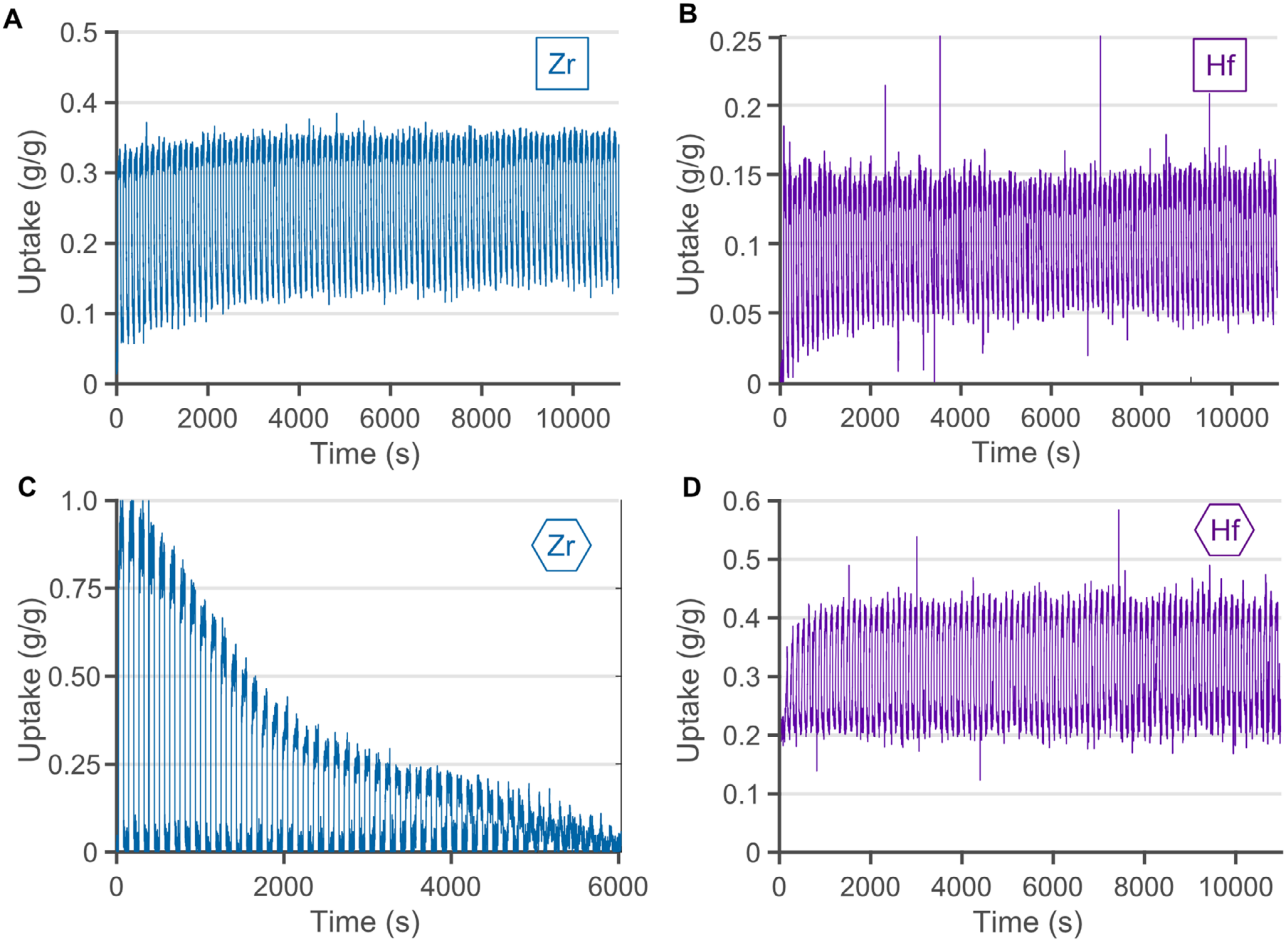
Kinetics of water uptake and Sorption Cycling. (A, B) Long‐term cycling behavior of (A) MOF‐801(Zr) and (B) MOF‐801(Hf) with a 60‐s interval between 0 and 40%RH. (C, D) Long‐term cycling behavior of (C) MOF‐808(Zr) and (D) MOF‐808(Hf) with a 60‐s cycling interval between 0 and 40%RH. MOF‐801(Zr) demonstrates robust cycling with stable residual water after 4000 s. In contrast, MOF‐808(Zr) exhibits a notable decline in water uptake capacity, culminating in framework collapse after about 40 cycles. Integration time in panels A+B: 1 s, C+D: 2 s, Excitation power: 33 mW/µm^2^.

MOF‐801(Zr) maintained stable performance throughout 100 cycles (200 min), although the desorption baseline gradually shifted upward during the first ∼50 cycles (Figure 7A). This persistent fraction of water—amounting to ∼25% of the initial capacity—varied slightly between particles but is consistent with water accumulating in local defect environments that cannot be emptied within 60 s of dry N_2_ flow. A similar trend was observed for MOF‐801(Hf), which remained structurally stable across 100 cycles (Figure 7B), but operated at approximately half the uptake of the Zr analogue. Notably, the initial rise in saturation capacity during the first ∼20 cycles of MOF‐801(Zr) suggests that residual DMF is slowly displaced during cycling, transiently opening additional pore volume.

In striking contrast, MOF‐808(Zr) exhibited a rapid decay in water uptake after only ∼6 cycles, with catastrophic loss of capacity by ∼40 cycles (Figure 7C). This behavior matches prior reports attributing the instability to capillary forces during water evacuation, which induce pore collapse in mesoporous Zr carboxylate frameworks [48]. Stabilization strategies such as halide‐assisted defect healing have been proposed [48], but these require modified synthesis protocols beyond the scope of this study. The collapse of MOF‐808(Zr) thus highlights a key limitation: although its ultrafast kinetics and high uptake make it an attractive AWH candidate, its cycling instability severely constrains practical deployment.

Guided by our analysis of computationally generated structures indicating reduced defectivity in Hf frameworks (Note S6), we decided to replace Zr with Hf in the MOF‐808 metal clusters to test whether replacing Zr with Hf increases structural robustness under cycling. Indeed, MOF‐808(Hf) remained stable throughout 100 cycles without signs of collapse (Figure 7D). Its adsorption and desorption kinetics were identical to those of MOF‐808(Zr), yet its maximum uptake per cycle was lower due to the strong Hf–DMF binding and reduced defect density discussed above. (Note S8.3). Water uptake at 40%RH per cycle decreased from ∼0.35 g g_MOF_^−1^ for MOF‐801(Zr) to ∼0.12 g g_MOF_^−1^ for MOF‐801(Hf), and from ∼0.7 g g_MOF_^−1^ for MOF‐808(Zr) to 0.4 g g_MOF_^−1^ for MOF‐808(Hf), respectively, due to the effects discussed previously. Nevertheless, the comparison between Zr and Hf variants clearly demonstrates that hafnium substitution stabilizes the framework against cycling‐induced degradation at the cost of reduced capacity.

Given these values, how much water could be theoretically collected? Following the adsorption and desorption times and the amount of water adsorbed at saturation (Figures 2, 5, and 7), we estimated the theoretical efficiency limits for active water harvesting (Table S13). Among the tested systems, MOF‐808(Zr) demonstrated the highest efficiency limit in the absence of interparticle condensation, reaching 375 L kg^−^^1^ h^−^^1^. The exceptionally fast adsorption and desorption kinetics of MOF‐808(Zr) make it a strong candidate for applications requiring rapid cycling. However, its instability during water sorption cycles, attributed to pore collapse [48], emphasizes the need for further optimization. Replacing Zr with Hf in MOF‐808 successfully stabilized the framework, yet at the expense of reduced water uptake capacity. Still, MOF‐808(Hf) achieved a theoretical uptake limit of over 100 L kg^−^^1^ h^−^^1^. MOF‐801(Zr) achieved a theoretical efficiency limit of 56 L kg^−^^1^ h^−^^1^ and stands out for its ability to adsorb water at relative humidity as low as 10%. In contrast, MOF‐801(Hf) displayed the slowest kinetics and lowest water uptake capacity among the tested materials, reaching only a theoretical efficiency limit of 11 L kg^−^^1^ h^−^^1^, though it adsorbs water at even lower relative humidities. The hygroscopic nature of both MOF‐801 materials makes them particularly suitable for use in arid environments, although their slower desorption kinetics clearly limits its cycling speed.

Remarkably, all four MOFs—when evaluated as isolated single crystals—exceed the water‐harvesting rates of state‐of‐the‐art passive devices (∼0.01–0.02 L kg^−^^1^ h^−^^1^ or 0.25–0.50 L kg^−^^1^ d^−^^1^) [49, 50, 51] by two to four orders of magnitude, and outperform even advanced active state‐of‐the‐art AWH systems (up to 0.5 L kg^−^^1^ h^−^^1^) [26, 52, 53] by factors of 22–750 under idealized laboratory conditions. These findings underscore the extraordinary intrinsic cycling capabilities of MOF single crystals in the absence of missing interparticle condensation and concomitantly fast adsorption and desorption times, while also highlighting the critical challenges posed by defect chemistry, solvent retention, and mesopore stability under realistic cycling environments.

## 3 Conclusion

This work examined how the choice of metal ions (Zr or Hf) and linker molecules, the presence and type of defects, as well as residual guest molecules, collectively impact water sorption and cycling in MOF‐801 and MOF‐808. We used a combination of single‐crystal Raman spectroscopy, ^1^H NMR, and DFT to elucidate particle‐to‐particle heterogeneity in water uptake, which is invisible in bulk measurements. In microporous MOF‐801, Zr‐based crystals absorb more water due to missing‐linker and missing‐cluster defects that increase the intrinsically available pore volume. Hf substitution, on the other hand, strengthens the interaction between node and linker. It suppresses defect formation and enhances the frameworks’ stability at the cost of increased DMF retention and reduced porosity. In mesoporous MOF‐808, the Zr‐based framework also achieves higher capacity and rapid kinetics but tends to collapse upon repeated cycling. Substituting Zr with Hf stabilizes the framework again, yet at the expense of lower operational pore volume due to stronger DMF coordination. Taken together, our results show that Zr‐based frameworks obtain higher uptake capacities through higher defect density, whereas Hf‐based materials are structurally more stable but absorb less. Furthermore, we found that residual DMF is a dominant, performance‐limiting factor. Conventional washing protocols often fail to completely remove guest molecules from the pores. As a result, materials prepared in different batches can be difficult to compare unless the extent of guest removal is carefully quantified. Control over solvent removal is therefore critical for a reproducible synthesis of MOFs as water‐sorbent materials. Overall, these findings offer practical guidance for the development of next‐generation water‐harvesting MOFs and emphasize the importance of carefully balancing metal–ligand bonding, defect engineering, and solvent removal to reach both high uptake and long‐term stability.

## Author Contributions

The E.P. conceived the research. E.P. supervised and designed the experiments. MOF samples were synthesized and characterized via SEM and XRD by O.Z. and J.A. under the supervision of S.W. MOF‐801 samples with different amounts of DMF were synthesized and characterized by J.T under the supervision of E.P. T.R. conducted the N_2_ sorption experiments. A.D. conducted DFT calculations under the supervision of L.G. J.T. performed the ^1^H NMR digestion assay. F.K. and J.T. contributed equally to the publication. F.K. and J.T. carried out optical microscopy and Raman measurements on SC‐MOF‐801 and MOF‐808 under the supervision of E.P. F.K., J.T., O.Z., J.A., and E.P. analyzed the data, conducted the statistical analysis, and designed the figures. F.K., J.T., O.Z., J.A., A.D., S.W., and E.P. wrote the manuscript. All authors approved the final version of the manuscript.

## Conflicts of Interest

The authors declare no conflict of interest.

## Supporting information




**Supporting File**: smll73636‐sup‐0001‐SuppMat.pdf.

## Data Availability

Cartesian coordinates of all optimized geometries are available as a ZIP archive containing the structural data generated from the computational modeling. Additional data that support the findings of this study are available from the corresponding authors upon reasonable request.
