# Supplementary material for: Unraveling Water Sorption in Single‐Crystal MOFs: Insights from Spectroscopy and Modeling on the Role of Structure, Composition, and Guest Molecules
Source: Small. 2026 May 6;22(34):e73636. doi: 10.1002/smll.73636 (PMC13275034; doi:10.1002/smll.73636)
Supplement: Supplementary file 1 — Supporting File: smll73636‐sup‐0001‐SuppMat.pdf. [file SMLL-22-e73636-s001.pdf]

Supplementary Information

Unraveling Water Sorption in Single-Crystal MOFs: Insights from Spectroscopy and Modeling on the Role of Structure, Composition and Guest Molecules

Jonas Tittel,[1,§] Fabian Knechtel,[1,§] Orysia Zaremba,[2] Andrea Darù,[3] Jacopo Andreo,[2] Laura Gagliardi,[3] Stefan Wuttke,[2,4] and Evelyn Ploetz[1,*]

1. Department of Chemistry and Center for NanoScience (CeNS), Ludwig-Maximilians-Universität München, Butenandtstr. 5-13, 81377 Munich, Germany
2. BCMaterials, Basque Center for Materials, UPV/EHU Science Park, 48940 Leioa, Spain
3. Department of Chemistry, Pritzker School of Molecular Engineering, and Chicago Center for Theoretical Chemistry, University of Chicago, Chicago, Illinois 60637, United States
4. Academic Centre for Materials and Nanotechnology, AGH University of Krakow, Krakow, 30-059 Poland

* Corresponding authors: evelyn.ploetz@lmu.de
§ These authors contributed equally to the publication.

TABLE OF CONTENT

SUPPLEMENTARY NOTE 1. CHARACTERIZATION TECHNIQUES 3
1.1. Scanning electron microscopy (SEM) 3
1.2. Powder X-ray diffraction (PXRD) 3
1.3. Gas sorption isotherms in Bulk 3
1.3.1. Low-temperature nitrogen sorption isotherms 3
1.4. *In-situ* Raman spectroscopy 4
1.4.1. Relative Humidity Unit 4
1.4.2. Humidity-dependent Raman-measurements 4
1.4.3. Data evaluation 5

SUPPLEMENTARY NOTE 2. MOF SYNTHESIS 8
2.1. Chemicals 8
2.2. Synthesis of MOF materials 8
2.2.1. Synthesis of MOF-801(Zr) single crystals. 8
2.2.2. Synthesis of MOF-801(Hf) single crystals. 8
2.2.3. Synthesis of MOF-808(Zr) single crystals. 9
2.2.4. Synthesis of MOF-808(Hf) single crystals. 9

SUPPLEMENTARY NOTE 3. MOF CHARACTERIZATION 10
3.1. Scanning electron microscopy 10
3.2. Powder X-ray diffraction 11
3.3. N_2 isotherms 11
3.4. Bright Field Microscopy 12

SUPPLEMENTARY NOTE 4. RAMAN SPECTRA IN DRY AND SATURATED HUMIDITY 13
4.1. Full Raman spectra 13
4.2. Fingerprint region 14

SUPPLEMENTARY NOTE 5. NMR MEASUREMENTS 16
5.1. NMR Digestion protocol 16
5.2. NMR spectra 16

SUPPLEMENTARY NOTE 6. DFT CALCULATIONS OF MOF-801 AND MOF-808 19
6.1. Uptake capacities 19
6.2. Structural confinement 22
6.3. Coordination free energies 23
6.4. Methods section: DFT calculations 25

SUPPLEMENTARY NOTE 7. SOLVENT BLOCKING: CONTROLLING RESIDUAL DMF IN MOF-801(ZR) 26
7.1 Synthesis and Washing Protocol 26
7.2 Bulk Characterization and Quantification of DMF 26
7.2.1. Structural characterization 26
7.2.2. Data evaluation and quantification of residual DMF by NMR 28
7.3 Quantification of residual DMF using vibrational spectroscopy 30
7.3.1 Raman Spectra of chemicals used during synthesis 30
7.3.2 Raman of batches after different numbers of washing cycles 31

SUPPLEMENTARY NOTE 8. KINETIC MEASUREMENTS 33
8.1. Readout of CCD Cameras 34
8.2. Measurement of kinetic series with long-term exposure 35
8.3. Comparison of SC kinetics for Zr- and Hf- based MOF-801 and MOF-808 36
8.4. Theoretical limits in water harvesting by Zr- and Hf- based MOF-801 and MOF-808 38

SUPPLEMENTARY REFERENCES 39

Supplementary Note 1. Characterization techniques

1.1. Scanning electron microscopy (SEM)

The surface morphology of the samples was examined using a Hitachi S-4800 field emission scanning electron microscope (FEG-SEM) operating at an accelerating voltage of 5 kV. Prior to imaging, the samples were coated with a 10 nm layer of gold using an Emitech K550x ion sputter to enhance conductivity and minimize charging effects during imaging.

1.2. Powder X-ray diffraction (PXRD)

X-ray powder diffraction patterns were obtained using a Panalytical X'pert PRO (Philips) diffractometer. The instrument operated at 40 kV and 40 mA in theta-theta configuration, employing a secondary monochromator with Cu K-α radiation (λ = 1.5418 Å) and a Pixel solid-state detector with an active length in 2θ - 3.347°. Data collection occurred over a 2θ range of 2 to 75° with a step size of 0.026° and a time per step of 700s at RT (total time 2h). To ensure consistent sample illumination, 1° fixed soller and divergence slits were employed, maintaining a constant volume of sample illumination.

1.3. Gas sorption isotherms in Bulk

1.3.1. Low-temperature nitrogen sorption isotherms

Nitrogen sorption isotherms were obtained using a Quantachrome Autosorb 1 at 77 K over a pressure range of p/p_0 = 0.001 to 0.98. Prior to sorption measurements, the samples underwent a 24-hour heating process at 120 °C under turbo-pumped vacuum conditions. The surface area was determined using the BET model within the pressure range of 0.05 to 0.3 p/p_0. Pore size distributions were analyzed using the QSDFT equilibrium model (desorption branch) with a carbon kernel for cylindrical pores. Connolly surfaces were generated employing an N_2-sized probe (r = 0.184 nm) at a grid interval of 0.025 nm.

1.4. *In-situ* Raman spectroscopy

Single MOF crystals were studied on a confocal Raman microscope. The setup operates with a continuous-wave diode laser (Cobolt SambaTM100 04-01 Series, Cobalt) with a maximum output of 100 mW at 532 nm. A dichroic (zet532/NIR, AHF Analysentechnik) separates the excitation from the detection path inside of the microscope body (TE 300, Nikon), while a water immersion objective (Plan APO VC 60x 1.2 NA, Nikon) focuses the laser on the sample. The Rayleigh scattered light is blocked by a notch (Chroma: zet532TopNotch) and a long-pass filter (Chroma RET537LP). Then, the signal is detected on a spectrometer (Kymera 328i, Oxford Instruments) equipped with an emCCD camera (iXon 897, Andor Solis). Raman spectra were measured with a 300 lines/mm grating without electronic gain, if not mentioned otherwise.

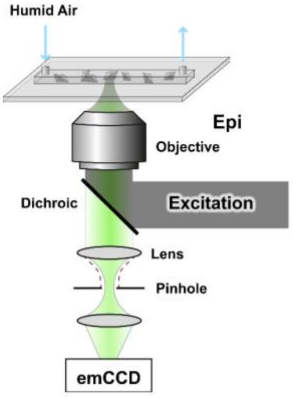

Supplementary Figure S1. Raman setup and experimental setup. *Schematic diagram depicting the confocal microscope and flowcell used for monitoring water sorption and harvesting performance at the single-crystal level. The flow-cell is connected to a Relative Humidity Unit, through which a nitrogen (N_2) stream containing water vapor at controlled humidity levels is supplied.*

1.4.1. Relative Humidity Unit

To carry out Raman spectroscopy *in situ* under defined humidity conditions, the microscope is equipped with a relative humidity unit (RHU).[1] The RHU has two inlets: (1) a dry N_2 stream (99,999%; Air Liquid Linde) and (2) a humidified N_2 stream. The humidified stream is enriched with water vapor by passing through a reservoir (500 mL) containing 400 mL of water. Mass flow controllers (MFC, D-6311-FGD-33-AV-99-0-S-DR, Wagner Mess- und Regeltechnik GmbH) regulate the flow rates of both gas streams, enabling precise adjustment of relative humidity levels. The RHU outlet directs gas to the microscope channel slides, in which MOF particles were examined (µ-Slide I Luer, Ibidi) on top of the confocal microscope (**Supplementary Figure S1**). The sample was positioned using a combined manual and XYZ piezo stage (BIO3.200; PiezoConcept). Spectra were recorded in the middle of the crystal if not stated otherwise. Data acquisition was achieved using the supplier's software Andor Solis for Imaging V4.30 (Oxford Instruments).

1.4.2. Humidity-dependent Raman-measurements

Single-crystal water isotherms. For SC measurements, vapor sorption was monitored by recording spectra between ~2300 and 4200 cm^{-1}. Before exposure to humid N_2, activated crystals were dried for at least 25 minutes in constant N_2 flow at 1 L / min. SC isotherms were obtained via Raman spectra taken under different humidity that was stepwise increased – with smaller steps in the beginning (0 to ~8% RH) and bigger intervals until saturation. To ensure a steady-state equilibrium after a humidity change, MOF crystals were exposed to the new condition for 6 minutes before spectrum acquisition. For the desorption, identical working steps

were applied in reverse. For SC isotherms, we chose a power density of ~33 mW/ µm^2 and an acquisition time per spectrum of 5x20s seconds.

Single-crystal sorption kinetics and cycling experiments. For kinetics or cycling experiments, MOF particles were first exposed to an N_2 stream for 20 minutes to ensure identical, dry starting conditions. The acquisition of the kinetic series started under N_2 flow. It was set as t_0 = 0 s when exposed to humid air. However, instead of measuring steady-state Raman spectra for different humidities, the MOFs were exposed to vapor at fixed relative humidities, and Raman spectra were recorded consecutively. By increasing the electronic gain of the emCCD camera to 150, the integration time could be decreased to 1 second for MOF-801 (2 seconds for MOF-808) per spectrum at a power density of ~33 mW/ µm^2. For cycling experiments, the Mass flow controllers were switched every 60 seconds between the lines to alternately expose the MOF dry N_2 (RH = 0%) and humid N_2 (RH = 40%). The respective measurement time points are chosen as the midpoint between the start and end time points of the spectrum acquisition (meaning the time frame from 0 – 1s is set as t = 0.5s).

1.4.3. Data evaluation

Raman spectra pre-processing and quantification. Guest molecules adsorbed to the framework were quantified via the Raman intensity A of the OH stretch vibration for water, using home-written scripts in Matlab2023b (Mathworks, Inc.). Raman spectra were pre-processed to compensate for baseline drifts, offset, and background noise. Cosmic ray artifacts were automatically removed by the data acquisition software (Andor Solis for Imaging V4.30; Oxford Instruments). Polynomial fitting was applied to address Rayleigh scattering and spurious background. The Raman intensity then reads

$$A_{Raman} = \int_{\tilde{\nu}_1}^{\tilde{\nu}_2} I_{corr}(\tilde{\nu}) d\tilde{\nu} \qquad \text{Eq. (1)}$$

with I_{corr} being the corrected Raman spectrum as a function of wavenumber $\tilde{\nu}$. The signal for water was identified between 3145 and 3665 cm^{-1}. Corrections were applied to address the spectral overlap between the OH stretch vibration of water and the CH stretch vibration of the framework, following established procedures (**Supplementary Figure S2**).[1] Gaussian fit functions of fixed shape were employed to model the OH and CH signature of water and the dry framework, respectively. The fit function was subsequently utilized to approximate the water content within the recorded Raman spectra and to assess the background contribution of water to the uncorrected signature of the CH stretch vibration.

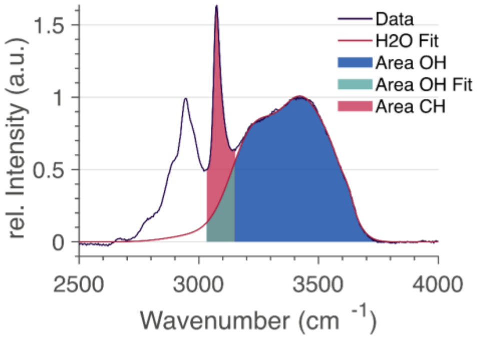

Supplementary Figure S2. Background correction of CH stretch vibration. *The Raman signature of, e.g., MOF-801(Zr) (black curve), is a linear superposition of the CH stretch signature of the dry framework and the OH stretch contributions from water molecules inside the framework. By modeling the OH stretch vibration of pure water and subtracting its contribution from the CH stretch signature, the isolated contribution of the CH stretch vibration can be determined (red area).*

The corrected intensity of the CH stretch vibration A_{CH} was employed as a measure for the amount of the bare MOF material within the confocal volume. We quantified the total amount of uptaken water, given in mass of guest m_{guest} per mass of MOF m_{MOF}, as following

$$\frac{m_{guest}}{m_{MOF}} = \frac{A_{guest}}{A_{CH}} \cdot \frac{\Gamma}{\rho_{MOF}} \qquad \text{Eq. (1)}$$

Here, ρ_{MOF} refers to the density of the MOF and Γ to an experimentally determined, model-supported scaling factor.[1] The scaling factor is calculated as follows

$$\Gamma = \frac{A_{CH}}{A_{H2O(l)}} \cdot \frac{1}{L_{Scatter}} \cdot \frac{1}{L_{Volume}(h)} \qquad \text{Eq. (2)}$$

The first term represents the ratio of the intensities between the CH stretch vibration of a dry MOF and the OH stretch vibration of liquid water. Assuming the MOF crystals were large enough to always fill the entire confocal volume, and the only interaction between the MOF and the Laser was Raman scattering, this would already give the calibration factor Γ. However, due to the effects just mentioned, additional correction factors are necessary. $L_{Scatter}$ accounts for the fact that Laser intensity within the MOF material is weaker than in water. Since water and the four MOF samples in this study do not adsorb in the visible/NIR range, this attenuation of the laser intensity is primarily attributed to scattering. To estimate the amount of light lost due to scattering within the framework, the transmission intensity of a broadband light source was measured through both the MOFs and water. $L_{scatter}$ is then given by the ratio of light transmitted through the MOF to the light transmitted through the water.

$$L_{Scatter} = \frac{I_{trans\ MOF}}{I_{trans\ H_2O}} \qquad \text{Eq. (3)}$$

Furthermore, we must correct for the fact that not all MOF crystals occupy the entire confocal volume in the Z direction. To account for this, we introduce a second correction factor, L_{Volume}. To calculate L_{Volume}, the intensity profile of the laser in Z direction within the confocal volume ($I_{confocal}(z)$) was measured by performing a Z-scan of a monolayer graphene surface and measuring the intensity of the graphene 2D peak at different positions along the Z-axis. The height (h) of the cubic MOF crystal was estimated by assuming a cubic shape and measuring

its dimensions in X/Y directions. The fraction of laser intensity interacting with the framework is then given by

$$L_{Volume}(h) = \frac{\int_{-h/2}^{h/2} I_{confocal}(z)\, dz}{\int_{-\infty}^{\infty} I_{confocal}(z)\; dz} \qquad \text{Eq. (4)}$$

With h = 0 referring to the height at maximum Raman signal inside MOF crystal. The scaling factors obtained by this method are summarized in **Supplementary Table S1**.

Table S1. Parameter to convert and quantify water uptake. *The error was calculated by the statistical error of the mean.*

**Sample**	**Density ρ_{MOF} (mg mm^3)**	**Literature**	$\boldsymbol{\Gamma_{Water}}$
**MOF-801(Zr)**	1.592	Furukawa (2014)[2]	0.096 ± 0.007
**MOF-801(Hf)**	2.105	Gu (2022)[3]	0.072 ± 0.005
**MOF-808(Zr)**	0.955	Furukawa (2014)[2]	0.096 ± 0.012
**MOF-808(Hf)**	1.278	Hu (2021)[4]	0.103 ± 0.012

Water quantification with respect to the molecular weight.

To accurately compare the water uptake performance of MOFs with different metal compositions, it is crucial to account for variations in their molecular weights, which are influenced by the atomic weight of the incorporated metals (e.g., zirconium vs. hafnium or guest molecules). While water uptake is conventionally reported in units of g(water) per g(MOF), this metric can be misleading when comparing materials with significantly different molecular weights. To address this, we converted the standard g/g format to g(water) per mol(MOF), which offers a more consistent basis for comparison by normalizing uptake to the molar basis of the material.

Following equation (1), the total molar amount of water uptake n_{Water}, normalized to the molar amount of MOF n_{MOF}, was calculated by multiplying equation (1) with the ratio of molecular masses M_{MOF} of the respective MOF system and dividing by the molecular mass of water M_{Water} (18.01 g/mol)

$$\frac{n_{Water}}{n_{MOF}} = \frac{M_{MOF}}{M_{Water}} \cdot \frac{m_{Water}}{m_{MOF}} \qquad \text{Eq. (5)}$$

The experimental molecular masses of MOFs derived from NMR digestion (see **Supplementary Note 5**) are summarized in **Supplementary Table S2**.

Table S2. Molecular Weight of studied MOF-801 and MOF-808 variants.

Sample	Unit formula	M_{exp} (g/mol)	Theoretical Formula	M_{theo} (g/mol)
MOF-801(Zr)	$Zr_6O_4(OH)_4(C_4O_4H_2)_5(CHO_2)_2(C_3NOH_7)_{0.1}$	1347.0	$Zr_6O_4(OH)_4(C_4O_4H_2)_6$	1363.7
MOF-801(Hf)	$Hf_6O_4(OH)_4(C_4O_4H_2)_5(CHO_2)_2(C_3NOH_7)_{0.9}$	1929.1	$Hf_6O_4(OH)_4(C_4O_4H_2)_6$	1887.3
MOF-808(Zr)	$Zr_6O_4(OH)_4(C_9O_6H_3)_2(CHO_2)_{3.64}(OH)_{2.36}(C_3NOH_7)_{0.30}$	1319.5	$Zr_6O_4(OH)_4(C_9O_6H_3)_2(CHO_2)_6$	1363.7
MOF-808(Hf)	$Hf_6O_4(OH)_4(C_9O_6H_3)_2(CHO_2)_{3.6}(OH)_{2.4}(C_3NOH_7)_{0.36}$	1846.4	$Hf_6O_4(OH)_4(C_9O_6H_3)_2(CHO_2)_6$	1887.3

Supplementary Note 2. MOF synthesis

2.1. Chemicals

All chemicals were purchased from commercial suppliers and used as received without further purification. *N,N*-Dimethylformamide (DMF, ≥ 99.9%; #DMFO-P0P), was obtained from LABKEM. Fumaric acid (#47910), benzene-1,3,5-tricarboxilic acid (#482749), hafnium (IV) oxychloride hydrate ($HfOCl_2 \cdot xH_2O$, 99.99%; #229652), zirconium oxychloride ($ZrOCl_2 \cdot 8H_2O$, 98%; #224316), acetone (≥ 99.5%; #179124), formic acid (> 96%; #F0507), and hydrochloric acid (37%; #258148) were ordered from Sigma Aldrich.

2.2. Synthesis of MOF materials

To ensure a consistent comparison between MOF-801 and MOF-808 structures with tuned metal composition (Zr vs. Hf), synthesis was performed using the same molar ratio of inorganic and organic components, with the metal source varied from $ZrCl_4$ to $HfCl_4$. Formic acid was employed as a modulator in all MOF syntheses. All samples underwent identical washing and activation procedures. For both bulk and single-crystal characterizations, activation was conducted at 120 °C under high vacuum for 24 h, as higher temperatures or prolonged times consistently led to crystal breakage. These conditions were also maintained for BET pre-measurement evacuation to account for potential guest molecules within the frameworks. After synthesis, all samples were characterized by SEM, PXRD, and N_2 sorption experiments.

2.2.1. Synthesis of MOF-801(Zr) single crystals.

Fumaric acid (27 mg, 0.23 mmol) and $ZrOCl_2 \cdot 8H_2O$ (75 mg, 0.23 mmol) were given into a 16 ml glass vial with a black cap. A mixture of DMF (11.67 mL) and formic acid (1.76 mL) was added and sonicated until the reagents were fully dissolved (about 20 minutes). The colorless solvent in the closed vial was heated at 120 °C for 2 days in a silica oil bath. Octahedral colorless crystals [$Zr_6O_4(OH)_4$ (fumarate)$_6$] were collected by centrifugation at 10000 rpm for 10 min after discarding the supernatant solution. The remaining material was washed with fresh DMF twice per day for 3 consecutive days and subsequently with fresh acetone twice per day for 3 days. The final sample was activated under a high dynamic vacuum (10^{-5} bar) at 120 °C for 24 h. The overall yield was 93% (49 mg).

2.2.2. Synthesis of MOF-801(Hf) single crystals.

Fumaric acid (27 mg, 0.23 mmol) and $HfOCl_2 \cdot xH_2O$ (95 mg, 0.23 mmol) were given into a 20 mL glass vial with a black cap. A mixture of 11.67 mL DMF and 1.76 mL formic acid was added and sonicated until the reagents were fully dissolved (about 20 minutes). The clear colorless solution in the closed vial was afterward heated at 120°C for 2 days in the silica oil bath. The supernatant solution was discarded and the sample was centrifuged at 10000 rpm for 10 min, before washing it with DMF twice per day for 3 days and subsequently with fresh acetone twice per day for 3 days. The final sample [$Hf_6O_4(OH)_4$ (fumarate)$_6$] was activated under a high dynamic vacuum (10-5 bar) at 120 °C for 24 h. The overall yield was 42% (31 mg).

2.2.3. Synthesis of MOF-808(Zr) single crystals.

$ZrOCl_2 \cdot 8\ H_2O$ (164 mg, 0.5 mmol) and 1,3,5-benzenetricarboxilic acid (107 mg, 0.5 mmol) were given into a 100 mL GL bottle. A mixture of 20 mL DMF and 20 mL formic acid was added and sonicated until the reagents were fully dissolved (about 20 minutes). The clear colorless solution was heated at 100°C for 7 days in an oven. The supernatant solution was discarded, and the sample was centrifuged at 10000 rpm for 10 min. Afterward, the remaining sample was washed with DMF twice per day for 3 following days and then with acetone twice per day for 3 days. The final sample $[Zr_6O_4(OH)_4\ (BTC)_2]$ was activated under a high dynamic vacuum (10^{-5} bar) and 120 °C for 24 h. The overall yield was 97% (111 mg).

2.2.4. Synthesis of MOF-808(Hf) single crystals.

$ZrOCl_2 \cdot xH_2O$ (270 mg, 0.5 mmol) and 1,3,5-benzenetricarboxilic acid (107 mg, 0.5 mmol) were put into a 100 ml GL bottle. A mixture of 20 mL DMF and 20 mL formic acid was added and sonicated until the reagents were fully dissolved (about 20 minutes). Next, the clear colorless solution was heated at 100°C for 7 days in the oven. The supernatant solution was discarded, and the sample was centrifuged at 10000 rpm for 10 min. Afterward, it was washed with DMF for 3 days, two times per day, and with acetone for 3 days, two times per day. The final sample $[Hf_6O_4(OH)_4\ (BTC)_2]$ was activated under a high dynamic vacuum (10^{-5} bar) at 120 °C for 24 h. The overall yield was 90% (142 mg).

Supplementary Note 3. MOF characterization

3.1. Scanning electron microscopy

The morphology of the *as*-prepared MOFs with SBUs based on zirconium and hafnium was analyzed by SEM (**Supplementary Figure S3 and S4**). All materials exhibit micrometer-sized octahedral crystals with ~ 10 µm and 20 µm in diameter for MOF-801 (squared icon; **Supplementary Figure S3**) for IBUs with zirconium and hafnium, respectively. MOF-808 crystals (hexagonal icon; **Supplementary Figure S4**) exhibit smaller sizes of around 4-5 µm (zirconium) and 2-3 µm (hafnium) in diameter.

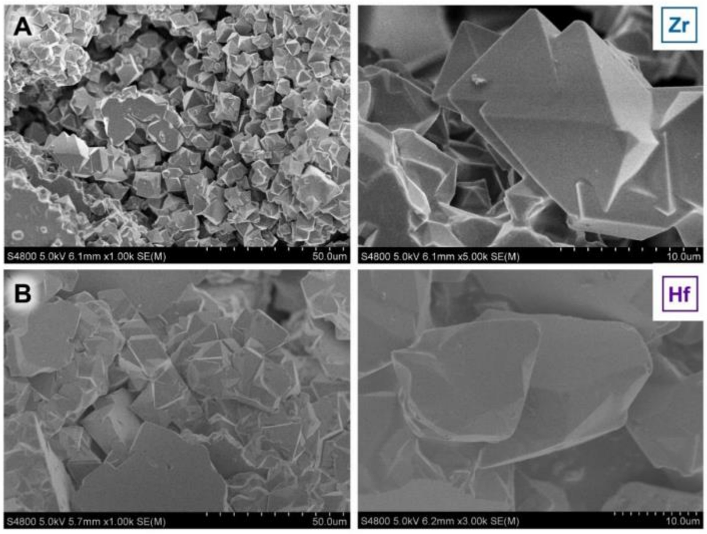

Supplementary Figure S3. SEM images. *SEM images of* ***(A)*** *MOF-801(Zr) and* ***(B)*** *MOF-801(Hf) at a resolution of 50 µm (left column) and 10 µm (right column). MOF-801(Zr) features octahedral crystals of about 10 µm, while MOF-801(Hf) exhibits intergrown particles of 20 µm in diameter and larger. Squared icon: MOF-801.*

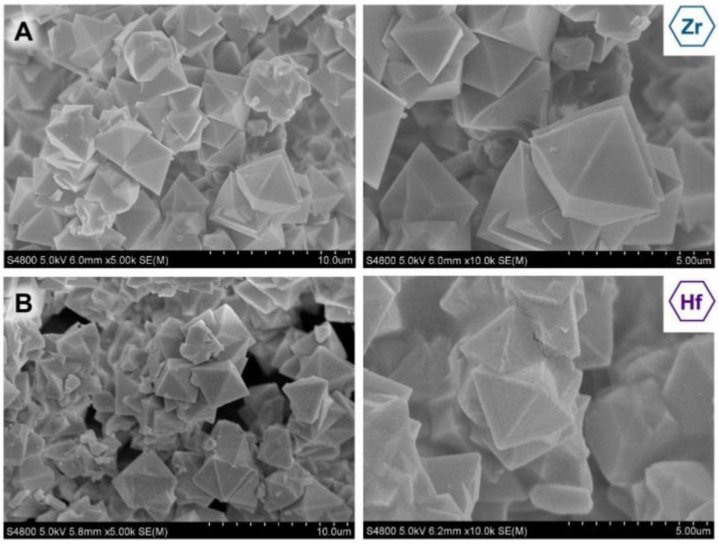

Supplementary Figure S4. SEM images. *SEM images of* ***(A)*** *MOF-808(Zr) and* ***(B)*** *MOF-808(Hf) at a resolution of 10 µm (left column) and 5 µm (right column). Both MOF materials show well-separated octahedral crystals of around 4-5 µm in size for MOF-808 (Zr) and around 2-3 µm in size for MOF-808(Hf). Hexagonal icon: MOF-808.*

3.2. Powder X-ray diffraction

PXRD patterns of MOF-801 and MOF-808 for Zr and Hf-based IBUs (**Supplementary Figure S5**) were obtained after activation under a high dynamic vacuum (10^{-5} bar) at 120 °C for 24 h. Simulated diffraction patterns for MOF-801 (CCDC 1002676) and MOF-808 (CCDC 1002672) are shown (black line) for comparison at the bottom of both panels.

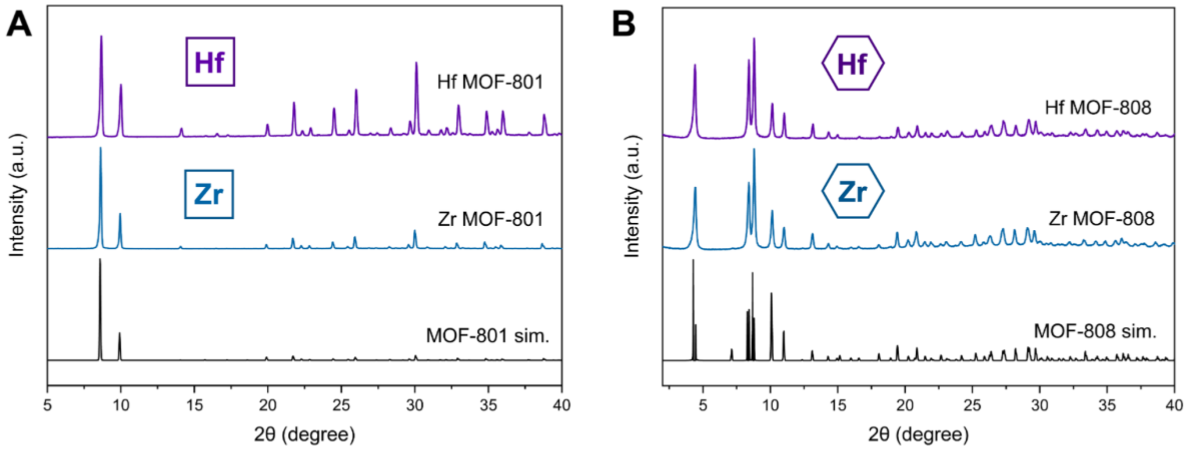

Supplementary Figure S5. Powder XRD. *PXRD pattern of **(A)** MOF-801 and **(B)** MOF-808 for MOFs with Hf (top; purple line) and Zr (middle; blue line). Simulated data is shown as a black line at the bottom. Legend: Square: MOF-801 crystals; Hexagon: MOF-808 crystals.*

3.3. N_2 isotherms

N_2 sorption isotherms were collected at 77 K with an Autosorb-1 analyzer (Quantachrome, USA) to evaluate the porosity of MOF-801 and -808 samples (**Supplementary Figure S6**).

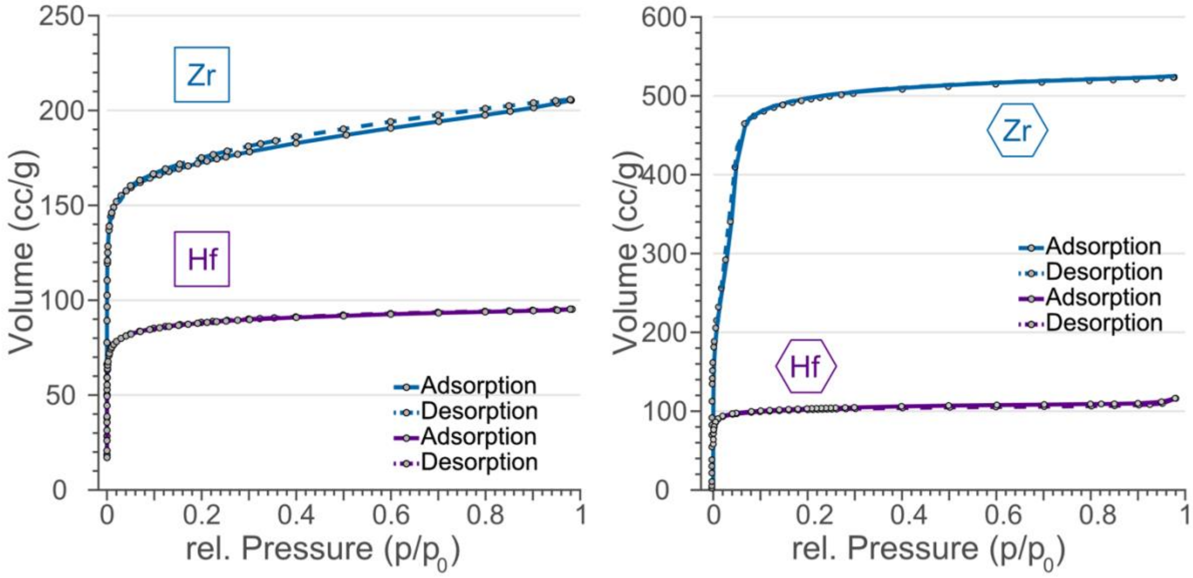

Supplementary Figure S6. N_2 isotherms. *Sorption isotherms were recorded for **(left)** MOF-801, and **(right)** MOF-808 made of zirconium (blue) and hafnium (purple) at 77K.*

Based on N_2 adsorption data, a Brunauer-Emmett-Teller (BET) calculation was performed on the BET range of 0.05 to 0.3 P/P_0. The extracted surface area is displayed in **Supplementary Table S3.**

Table S3. Pore size distribution. *BET surfaces, Langmuir surface areas, pore width, N_2 uptakes, and total pore volumes for all four MOF samples.*

**Sample**	**BET surface area ($m^2\ g^{-1}$)**	**N_2 uptake ($cm^3\ g^{-1}$ STP) §**
**MOF-801(Zr)**	656	205.4
**MOF-801(Hf)**	341	95.2
**MOF-808(Zr)**	1245	525.9
**MOF-808(Hf)**	404	116.2

§ measurement was taken at P/P_0=0.98.

3.4. Bright Field Microscopy

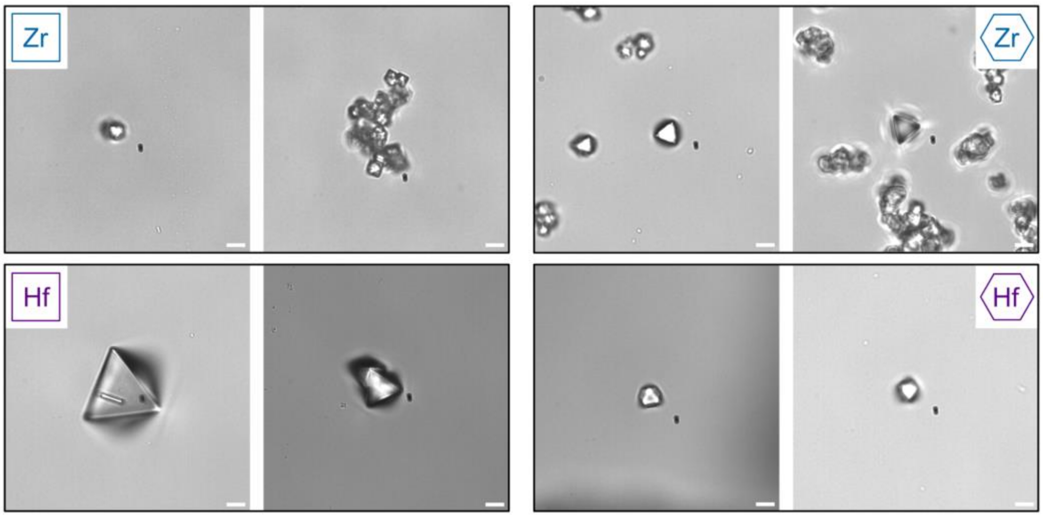

Supplementary Figure S7. Brightfield images of MOF-801 and MOF-808 based on zirconium and hafnium used to derive SC isotherms. *Single MOFs crystals have a diameter of around ~3 µm for MOF-801(Zr), ~10 µm for MOF-801(Hf), ~5 µm for MOF-808(Zr) and 4-5 µm for MOF-808(Hf). Scale bar: 5 µm.*

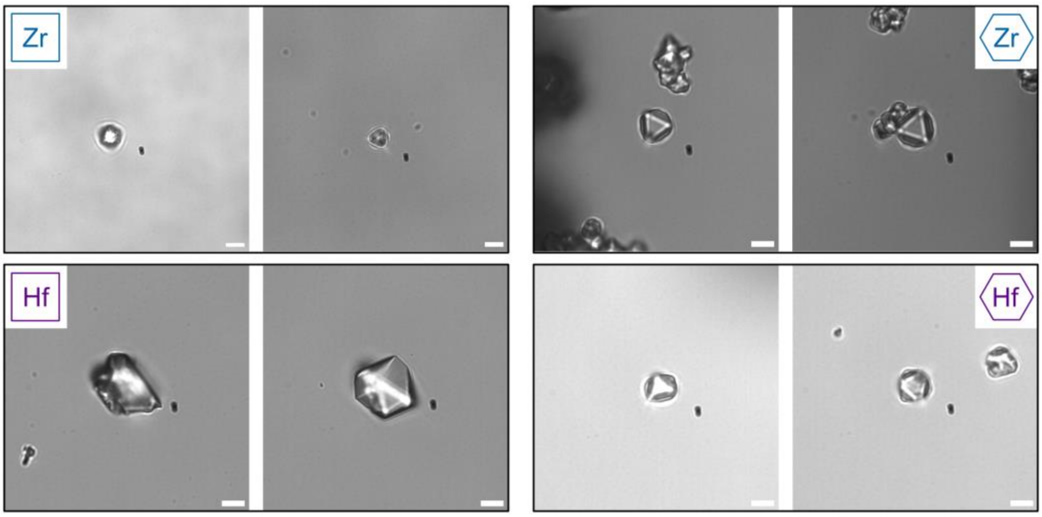

Supplementary Figure S8. Brightfield images of MOF-801 and MOF-808 based on zirconium and hafnium used for cycling studies. *Single MOFs crystals have a diameter of around ~3 µm for MOF-801(Zr), ~10 µm for MOF-801(Hf), ~5 µm for MOF-808(Zr) and 4-5 µm for MOF-808(Hf). Scale bar: 5 µm.*

Supplementary Note 4. Raman spectra in dry and saturated humidity

4.1. Full Raman spectra

Raman spectra of MOF-801 are dominated by the spectral signatures of the fumaric acid inside the framework (**Supplementary Figure S9**).[5] Additional resonances at 2939, 2867, 1676 and 866 cm^{-1} are indicative of residual DMF molecules incorporated during synthesis. Contributions due to water can be observed via its OH stretch vibration around 3300 cm^{-1}, the asymmetric OH bending mode around 1600 cm^{-1} and the liberational modes below 500 cm^{-1}. Contribution by the metal-clusters are visible below 900 cm^{-1} and as µ3-OH stretch vibration at 3682 cm^{-1}.

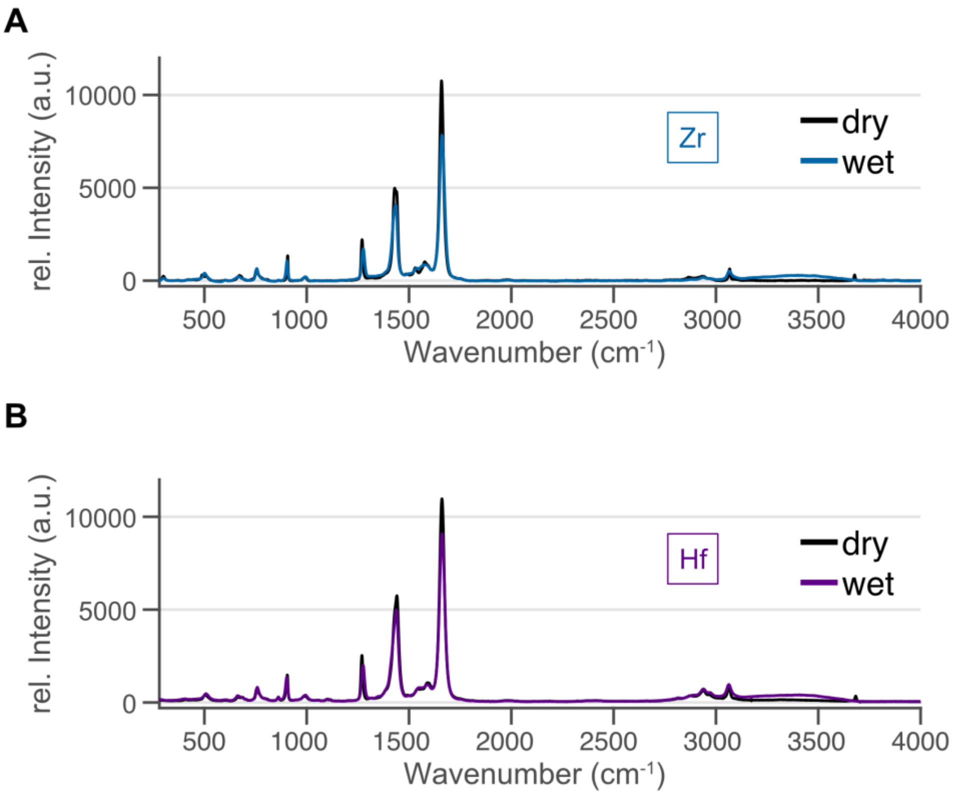

Supplementary Figure S9. MOF-801*. Raman spectra of* ***(A)*** *MOF-801(Zr) and* ***(B)*** *MOF-801(Hf) in dry state and after exposure to 40 %RH water vapor. Experimental settings: laser power 33 mW/µm², exposure time 5x25 s, grating 1200 lines/mm.*

Similarly, Raman spectra of MOF-808 are dominated by the spectral signatures of the trimesic acid within the framework (**Supplementary Figure S10**). Resonances at 2939, 2867, 1676 and 866 cm^{-1} are due to residual DMF molecules incorporated into the framework during synthesis.

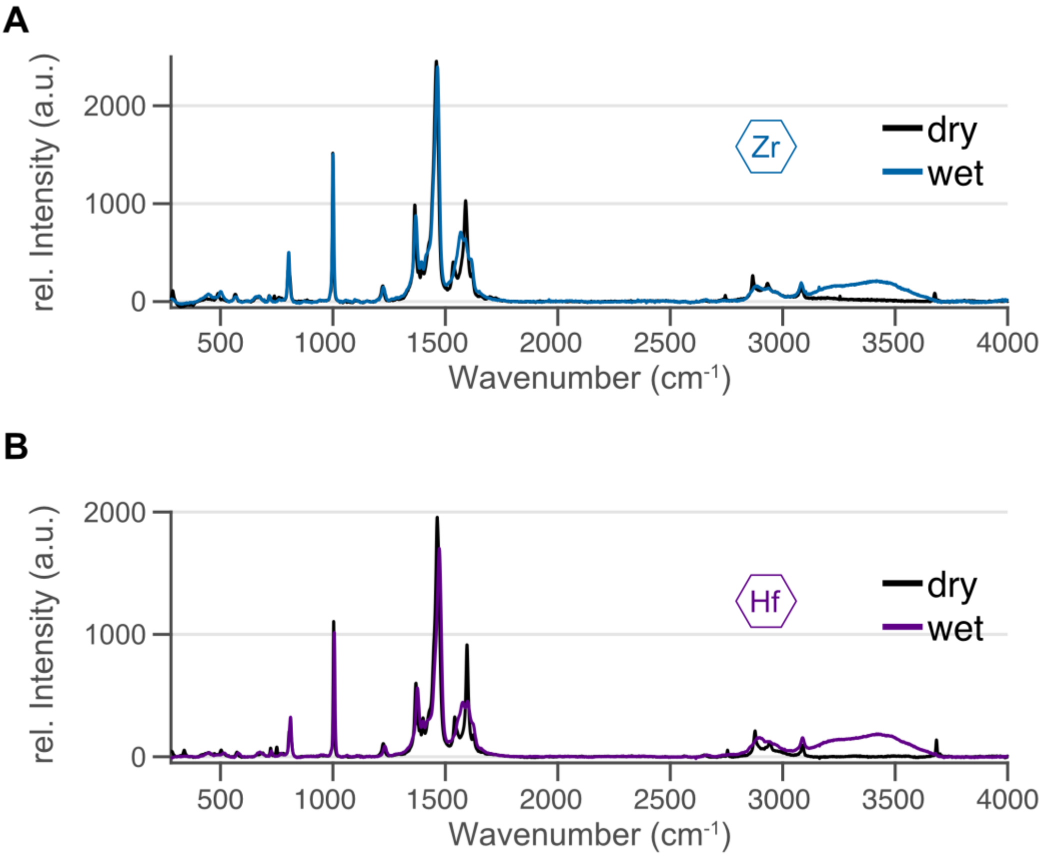

Supplementary Figure S10. MOF-808. *Raman spectra of* ***(A)*** *MOF-808(Zr) and* ***(B)*** *MOF-808(Hf) in dry state and after exposure to 40 %RH water vapor. Experimental settings: laser power 33 mW/µm^2, exposure time 5x25 s, grating 1200 lines/mm.*

4.2. Fingerprint region

The inorganic building unit (IBU) in MOF-801 and MOF-808 consist of a metal-oxide cluster. Under optimal conditions, the IBU is composed of a metal center (Zr or Hf) coordinated with oxygen atoms and hydroxyl groups. To calculate the molecular weight of the IBU when changing from zirconium to hafnium, we considered $Zr_6O_4(OH)_4$ or $Hf_6O_4(OH)_4$ clusters in these frameworks:

- Zirconium (Zr) has an atomic weight of approximately 91.22 g/mol.
- Hafnium (Hf) has an atomic weight of approximately 178.49 g/mol.

When substituting zirconium by hafnium in MOF-801 and MOF-808, the molecular weight of the inorganic building unit roughly doubles with a

- Molecular weight of the Zr-based IBU ($Zr_6O_4(OH)_4$): 679.36 g/mol
- Molecular weight of the Hf-based IBU ($Hf_6O_4(OH)_4$): 1,202.98 g/mol

Since vibrational frequency in Raman spectroscopy are inversely related to the square root of the reduced mass, the increase in mass results in lower vibrational frequencies for Raman peaks associated with the M-O (metal-oxygen) stretching vibrations. The Zr-O stretch appears in the Raman spectrum around 450 cm^{-1}. Substituting Zr with Hf leads to a downward shift in this peak, as expected.

Yet, when it comes to the linker-related Raman transitions, the substitution has a more subtle effect compared to the direct metal-oxygen vibrations (**Supplementary Figure S11**). Although zirconium and hafnium belong to the same group in the periodic table, hafnium has a slightly smaller ionic radius and forms slightly stronger bonds. As known from the literature, a substitution in the metal node of MOFs from zirconium to hafnium results in shorter distances in the μ^3-OH and M-OC modes.[6] Hence, vibrations related to the interaction between the metal center and the carboxylate groups of the linkers feel the difference between Zr and Hf. Blue shifts in Raman bands translate into vibrational modes with higher energy, which can be attributed to the greater acidity of Hf ions. Raman peaks originating from vibrations of aromatic linker molecules exhibit a similar behavior due to the smaller pore sizes and smaller crystal lattices (bonds are more contracted).

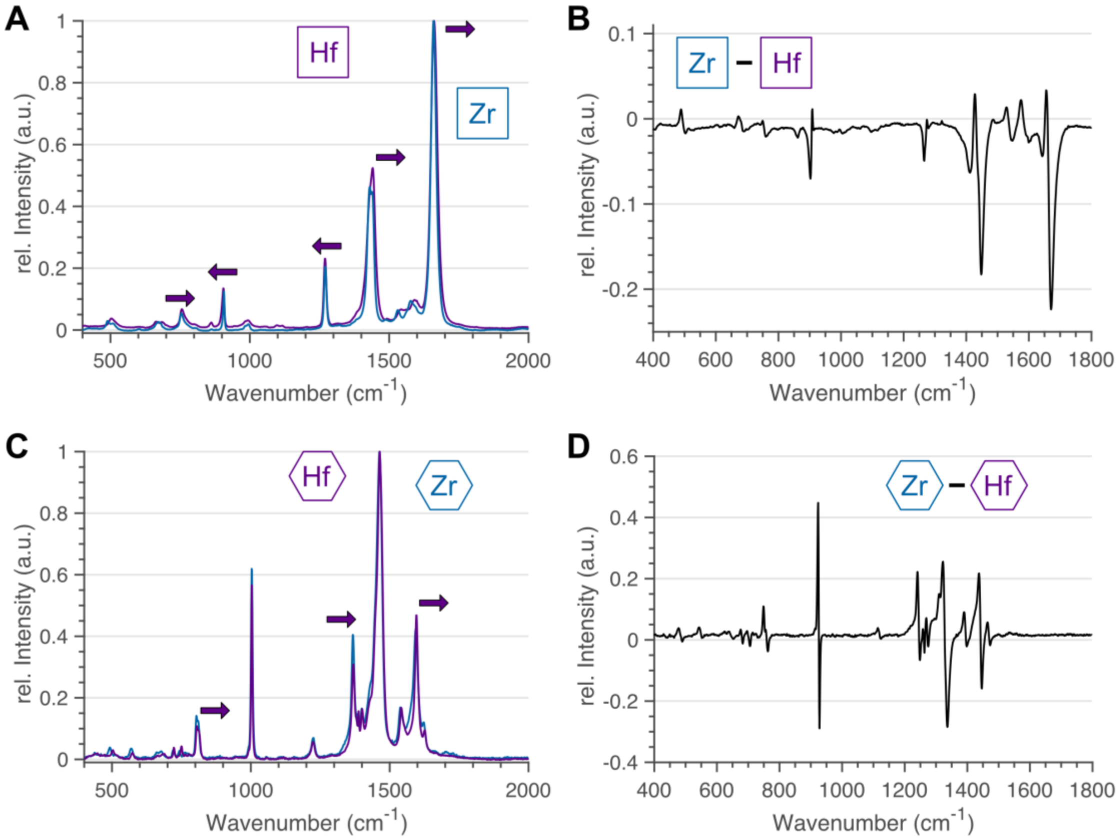

Supplementary Figure S11. Impact of Hafnium in MOF-801 and MOF-808. (A) *Fingerprint spectrum of MOF-801 with Zr- and Hf-IBU.* ***(B)*** *Difference spectrum for MOF-801.* ***(C)*** *Fingerprint spectrum of MOF-808 with Zr- and Hf-IBU.* ***(D)*** *Difference spectrum for MOF-808. The spectra are dominated by vibrational modes of the linker in the fingerprint region. Differences in metal composition are well-visible below 1000 cm^{-1}. The exposure time was 5x25s per grating step at 33 mW/µm^2.*

Supplementary Note 5. NMR Measurements

During the synthesis of MOF-801 and MOF-808, several guest molecules - including modulator and solvent molecules - remain within the materials. To investigate their presence and quantity, we performed NMR spectroscopy on digested MOF samples. The following Supplementary Note describes the methodology for the NMR analysis, the ^{1}H NMR spectra obtained for all MOF-801 and MOF-808 samples, and determined the amounts of guest molecules.

5.1. NMR Digestion protocol

For digesting MOF-801 or MOF-808, approximately 3 mg sample was added to a digestion solution consisting of 24 mg CsF, 450 µL DMSO-d6, and 250 µL D_2O. The mixture was vortexed to ensure a thorough dispersion of the sample, followed by sonication for 5 minutes to promote the dissolution and breakdown of the material. This process was repeated twice to ensure complete digestion of the MOF before transferring it into an NMR tube for further analysis. High-resolution ^{1}H-NMR data were collected on a Bruker 800 MHz NMR spectrometer. The experiments were conducted using a standard zg30 pulse sequence. Data were collected at a temperature of 298 K (room temperature) using DMSO-d6/D_2O as the solvent, with locking on the deuterium signal. The spectral width was set to 13.945 kHz, and the number of points in the FID was 131072. NMR data was processed using MestReNova Version 15.1.0. The spectra were referenced to the DMSO solvent peak at 2.5 ppm.

5.2. NMR spectra

The NMR spectra of all four MOF systems display characteristic peaks corresponding to the residual molecules from the synthesis process (besides the resonances of the organic linkers). The linker molecules in both MOF-801 and MOF-808 are identified at around 6.5 ppm (fumaric acid) and 8.5 ppm (aromatic ring, trimesic acid), respectively. Resonances from DMF (dimethylformamide) appear at δ ~2.75 ppm (CH_3 group), δ ~2.85 ppm (CH_3 group), and δ ~7.8 ppm (proton signal of the acyl group), although these peaks are weaker than those from the linkers. The modulator molecule, formic acid (FA), shows a resonance at around 8 ppm, which is characteristic of the proton signal from the carboxyl group.

To determine the degree of guest molecules, we calculated the ratio of the peak integrals corresponding to the guest molecules—specifically, the proton signal from the acyl group at 7.8 ppm (for DMF) or the carboxyl group around 8 ppm (for FA)—to the peak integrals of the respective linkers, which appear at approximately 6.5 ppm (for MOF-801) and 8.5 ppm (for MOF-808). The NMR spectra are shown in **Supplementary Figures S12** to **S15**.

Table S4. Modulator content in both MOF-801 and MOF-808 variants.

	**Evaluation**	**Linker / FA**
**MOF-801(Zr)**	1H (fumaric acid) / 1H (formic acid)	1 / 0.21
**MOF-801(Hf)**	1H (fumaric acid) / 1H (formic acid)	1 / 0.20
**MOF-808(Zr)**	1H (trimesic acid) / 1H (formic acid)	1 / 0.62
**MOF-808(Hf)**	1H (trimesic acid) / 1H (formic acid)	1 / 0.60

While the amounts of incorporated modulator molecules in both MOF-801 and MOF-808 variants are approximately identical (**Supplementary Table S4**), their NMR spectra reveal significant differences in the DMF content within the materials (**Supplementary Table S5**). The NMR spectrum of MOF-801 shows a low concentration of DMF molecules relative to the linker molecules, with DMF being present in a much smaller proportion. In contrast, Hf-MOF-801 exhibits a significantly higher DMF content, indicating approximately ten times more DMF in the Hf-based framework compared to the Zr variant. For MOF-808, there is a slight increase in DMF content (~20%) in Hf-MOF-808 compared to MOF-808(Zr), but the difference is much smaller than that observed between the MOF-801 variants. These results suggest that the DMF content is notably higher in MOF-801(Hf), which could influence its pore volume and uptake behavior compared to MOF-801(Zr).

Table S5. DMF content in both MOF-801 and MOF-808 variants.

	Evaluation	Linker / DMF	Linker / Formic acid
MOF-801(Zr)	1H (fumaric acid) / 1H DMF	1 / 0.02	1 / 0.42
MOF-801(Hf)	1H (fumaric acid) / 1H DMF	1 / 0.18	1 / 0.4
MOF-808(Zr)	1H (trimesic acid) / 1H DMF	1 / 0.15	1 / 1.86
MOF-808(Hf)	1H (trimesic acid) / 1H DMF	1 / 0.18	1 / 1.8

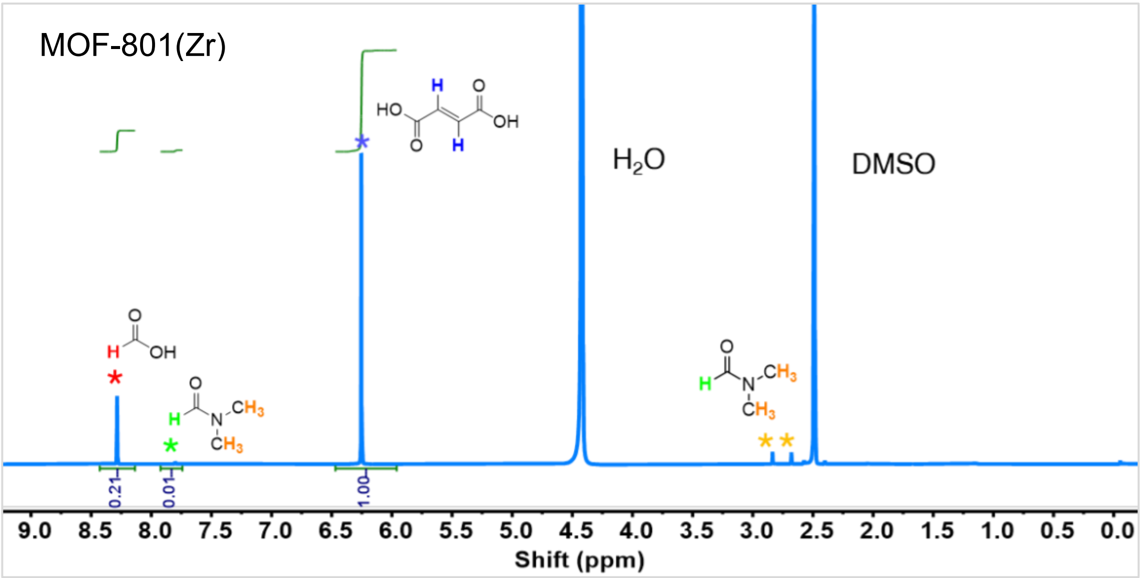

Supplementary Figure S12. ***^{1}H NMR (DMSO/D_2O, 800MHz) spectrum of digested MOF-801(Zr) samples.*** *MOF-801 samples based on zirconium were digested by CsF and dissolved in a DMSO-d6/D_2O mixture. Besides the characteristic peaks of the solvents, the NMR spectra reveal signatures of fumaric acid, as well as DMF and formic acid.*

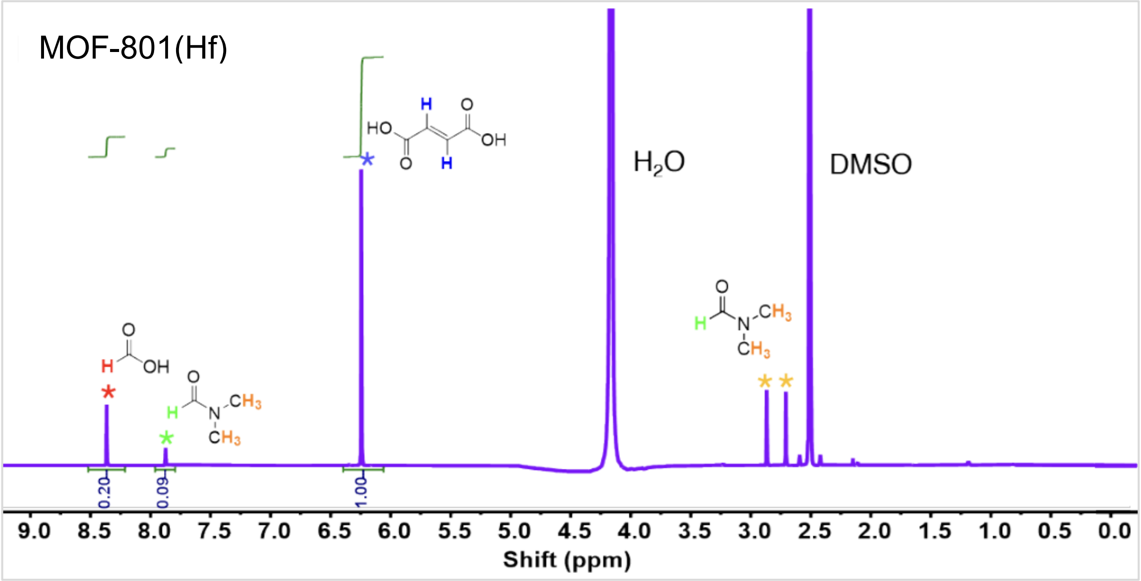

Supplementary Figure S13. ***^{1}H NMR (DMSO/D₂O, 800MHz) of digested MOF-801(Hf) samples.*** *MOF-801 samples based on zirconium were digested by CsF and dissolved in a DMSO-d6/D₂O mixture. Besides the characteristic peaks of the solvents, the NMR spectra reveal signatures of fumaric acid, as well as DMF and formic acid.*

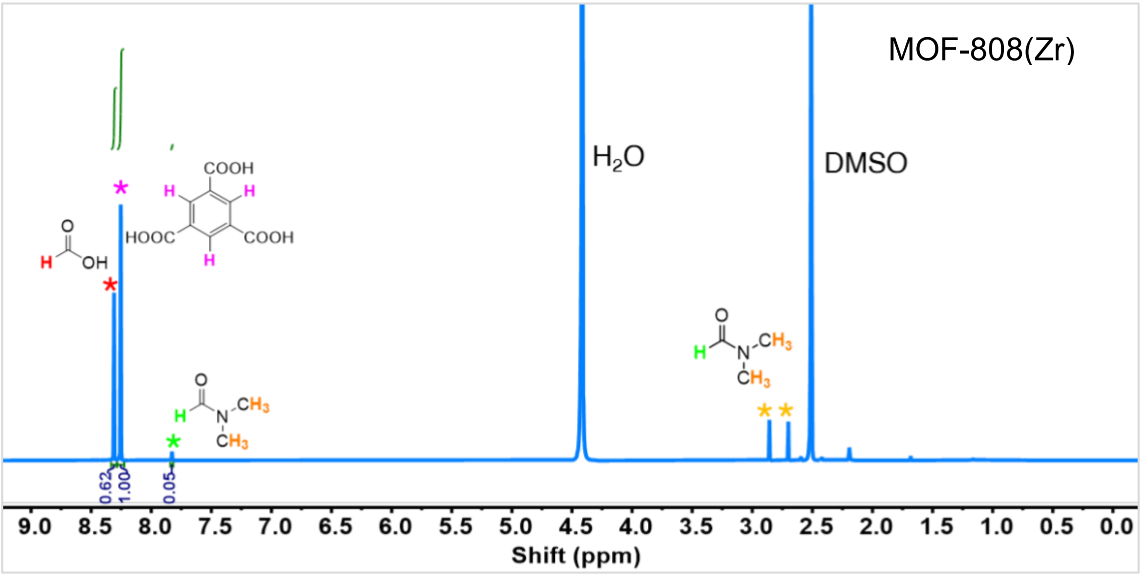

Supplementary Figure S14. ***^{1}H NMR (DMSO/D₂O, 800MHz) of digested MOF-808(Zr) samples.*** *MOF-808 samples were digested by CsF and dissolved in a mixture of DMSO-d6/D₂O. Besides the characteristic peaks of the solvents, the NMR spectra reveal signatures of trimesic acid, as well as DMF and formic acid.*

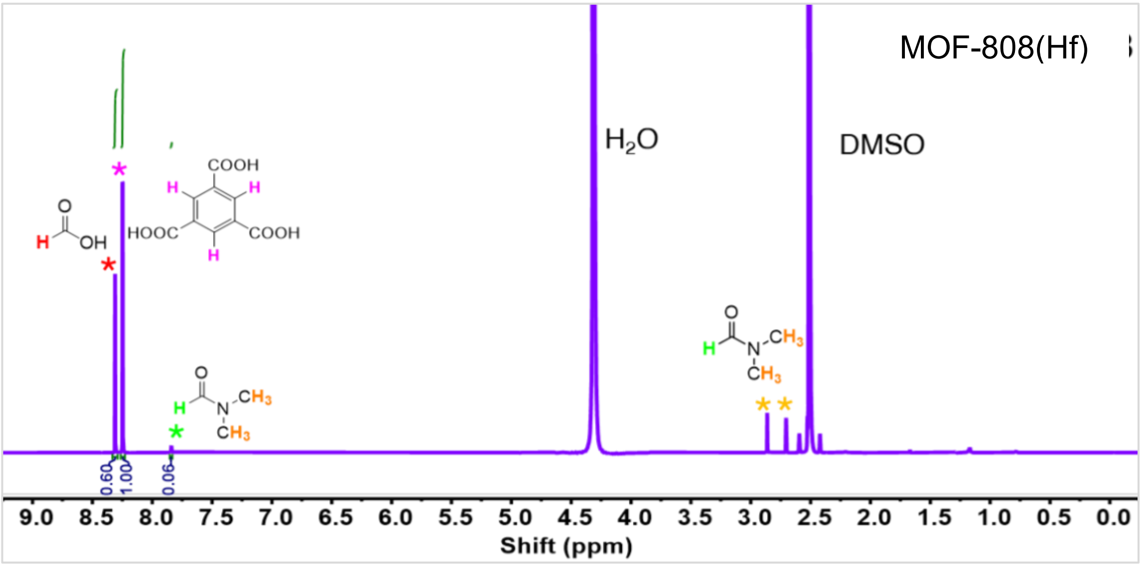

Supplementary Figure S15. ***^{1}H NMR ($DMSO/D_2O$, 800MHz) of digested MOF-808(Hf) samples.*** *MOF-808 samples were digested by CsF and dissolved in a mixture of DMSO-d6/D_2O. Besides the characteristic peaks of the solvents, the NMR spectra reveal signatures of trimesic acid, as well as DMF and formic acid.*

Supplementary Note 6. DFT Calculations of MOF-801 and MOF-808

The insertion of hafnium (Hf) into MOF-801 and MOF-808 has been observed to result in a decrease in water uptake capacity compared to their zirconium (Zr) counterparts, as well as a different number of defects. The following section first addresses the defect-dependent pore volumes, followed by structural insights gained from simulated PXRD data, which are compared to experimental results. Finally, the energetic contributions of adsorption between water molecules and various sites or guest molecules within the MOF-801 and MOF-808 frameworks are modeled using DFT calculations.

6.1. Uptake capacities

First, DFT calculations were performed on both pristine and defective MOF structures to calculate the available pore volumes (AV, see Computational Details Section; **Supplementary Figure S16**). These calculations were carried out using periodic DFT (PBE-D3BJ VASP), with no solvent present in the pores. The AV values of the computed structures were compared with the experimental maximum uptake from isotherms. In the case of MOF-801 (**Supplementary Figure S16a-b**) we found that the experimental AV for MOF-801(Zr) was larger than the DFT simulated value (0.373 g/g vs 0.291 g/g; **Supplementary Table S6**), which suggests that defects, such as missing nodes or linkers, might contribute to the higher uptake. In contrast, MOF-801(Hf) exhibited the opposite trend, with the experimental AV being smaller than the calculated value (0.138 g/g vs 0.203 g/g). This discrepancy in MOF-801(Hf) suggests that the presence of solvent molecules, occupying the pores or coordinating to the nodes, could be responsible for the lower experimental uptake.

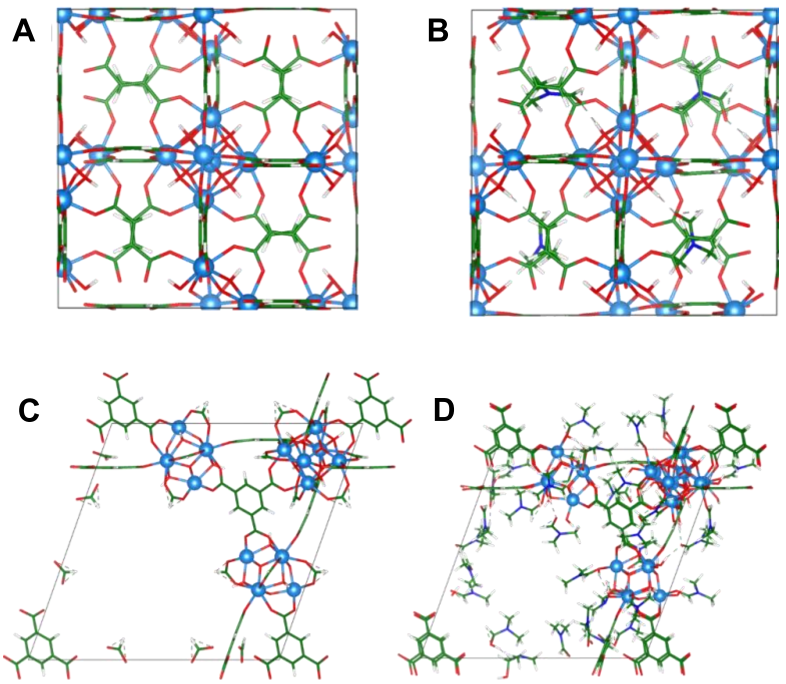

Supplementary Figure S16. Framework structures of MOF-801 and MOF-808 shown with different types of defects. *From left to right: pristine networks of A) MOF-801, and C) MOF-808; defective networks of B) MOF-801, and D) MOF-808 with missing linkers and added DMF molecules. Elements are colored: hydrogen atoms (white), oxygen atoms (red), carbon atoms (green), and zirconium or hafnium (cyan).*

***Table S6. Available pore volume in g/g of experimental in pristine MOF-801 without and with missing linkers interacting with water*.**

	**Exp.**	**No defects**	**2 linkers miss. H_2O/OH**	**4 linkers miss. H_2O/OH**	**6 linkers miss. H_2O/OH**
**MOF-801(Zr)**	**0.374**	0.291	0.301	**0.313**	**0.324**
**MOF-801(Hf)**	**0.138**	0.203	0.210	0.218	0.224

Missing linkers from MOF-801(Zr/Hf) is discussed in the main text. On the other hand defects such missing node were also accounted for all studied MOFs. Indeed, calculations involving missing linker defects alone are not sufficient to fully explain the measured uptake behavior. To address this, we next investigate the impact of missing nodes on the pore volume and uptake capacity. This is achieved by constructing a model without a node. We found that the removal of just one node in both MOF-801(Zr) and MOF-801(Hf) already resulted in a large increase in available volume that exceeds the experimentally observed uptake capacities of both systems (**Supplementary Table S7**). Even when up to six DMF molecules were included to simulate the solvent effects, the calculated AV still did not fully align with the experimental data. This discrepancy suggests that for MOF-801(Zr), a combination of unit cells with missing linkers and missing nodes must be present to reconcile the measured water isotherms.

Table S7. Available pore volume in g/g of experimental in pristine MOF-801 without and with one missing node in the presence and absence of additional DMF guest molecules.

	Exp.	Missing node H_2O/OH	Missing node H_2O/OH 1 DMF	Missing node H_2O/OH 2 DMF	Missing node H_2O/OH 4 DMF	Missing node H_2O/OH 6 DMF
**MOF-801(Zr)**	**0.374**	0.576	0.556	0.531	0.492	0.454
**MOF-801(Hf)**	**0.138**	0.400	0.388	0.372	0.347	0.321

In contrast, for MOF-801(Hf), contributions of missing node defects need to be rare, as their contribution can hardly be compensated. In this case, MOF-801(Hf) appears to be primarily influenced by the presence of DMF molecules replacing the linkers (see **Supplementary Table S8**), or by DMF solvents coordinated to the OH group of the node.

Table S8. Available pore volume in g/g of experimental in pristine MOF-801 without and with one missing linkers in the presence and absence of additional DMF guest molecules.

	Exp.	2 linkers miss. H_2O/OH 2 DMF	4 linkers miss. H_2O/OH 4 DMF	2 linkers miss. OH 4 DMF	3 linkers miss. OH 6 DMF	4 linkers miss. OH 8 DMF	No defects 4 DMF to node OH
**MOF-801(Zr)**	**0.374**	0.270	—	0.248	0.209	—	—
**MOF-801(Hf)**	**0.138**	0.189	0.178	0.170	**0.148**	0.090	**0.148**

A similar study was performed on MOF-808 (**Supplementary Figure S16c-d**), which, as in the case of MOF-801(Hf), shows that defects are less likely to occur. Instead, the OH/DMF pair coordinates with the nodes, replacing the formate modulator. This substitution results in a structure that better matches experimental results. As summarized in **Supplementary Table S9**, the presence of either 24 or 25 DMF molecules in the pores closely aligns with the experimental available volume (AV). In contrast, MOF-808(Zr) cannot be fully described by the removal of linkers, replaced by OH/DMF, as the computed AV values do not perfectly match the experimental values derived from water isotherms. This discrepancy equally suggests that a combination of defects may be present, as proposed in the case of MOF-801(Zr).

Table S9. Available pore volume in g/g of experimental in pristine MOF-808 without and with 24 formate missing substituted by OH/DMF, and extra DMF solvent in the tetrahedral pores.

	Exp.	No defects	24 formate missing 24 OH/DMF	24 formate missing 1 DMF+ 24 OH/DMF	24 formate missing 2 DMF+ 24 OH/DMF
**MOF-808(Zr)**	**0.778**	0.871	—	—	—
**MOF-808(Hf)**	**0.396**	0.619	0.397	0.391	0.383
			**1 linker miss. 1 DMF + 24 OH/DMF**	**2 linkers miss. 1 DMF + 24 OH/DMF**	**3 linkers miss. 1 DMF + 24 OH/DMF**
**MOF-808(Zr)**	**0.778**	0.871	0.530	0.523	0.458
**MOF-808(Hf)**	**0.396**	0.619	—	—	—

6.2. Structural confinement

Based on our simulations of the water isotherms and the identification of potential structures for MOF-801(Zr) and MOF-801(Hf), including contributions from defects and guest molecules, we further analyzed their corresponding PXRD spectra. As seen in **Supplementary Note 3.2**, both Hf- and Zr-based MOF systems exhibit nearly identical PXRD spectra. They share an identical topology with minimally smaller pore sizes for Hf-based systems. When compared to simulations, we found that node-deficient MOF structures should exhibit peaks at 5 and 7.5 degrees, which are not observed in the experimental PXRD pattern (**Supplementary Figure S17A**). Since the experimental powder diffractograms do not show additional peaks nor peak broadening, the defectivity introduced by missing nodes has no periodic organization in the crystal. Moreover, it does not strongly influence the cell parameters. Upon comparing the PXRD patterns of MOF-801(Zr) and MOF-801(Hf) with various defect configurations, including missing linkers and DMF coordination, we observed that the simulated spectra for MOF-801(Hf) with 2 and 3 missing linkers replaced by OH/DMF residues (**Supplementary Figure S17B-C**) and with DMF molecules coordinated to the node (**Supplementary Figure S17D**) showed much better alignment with the experimental data.

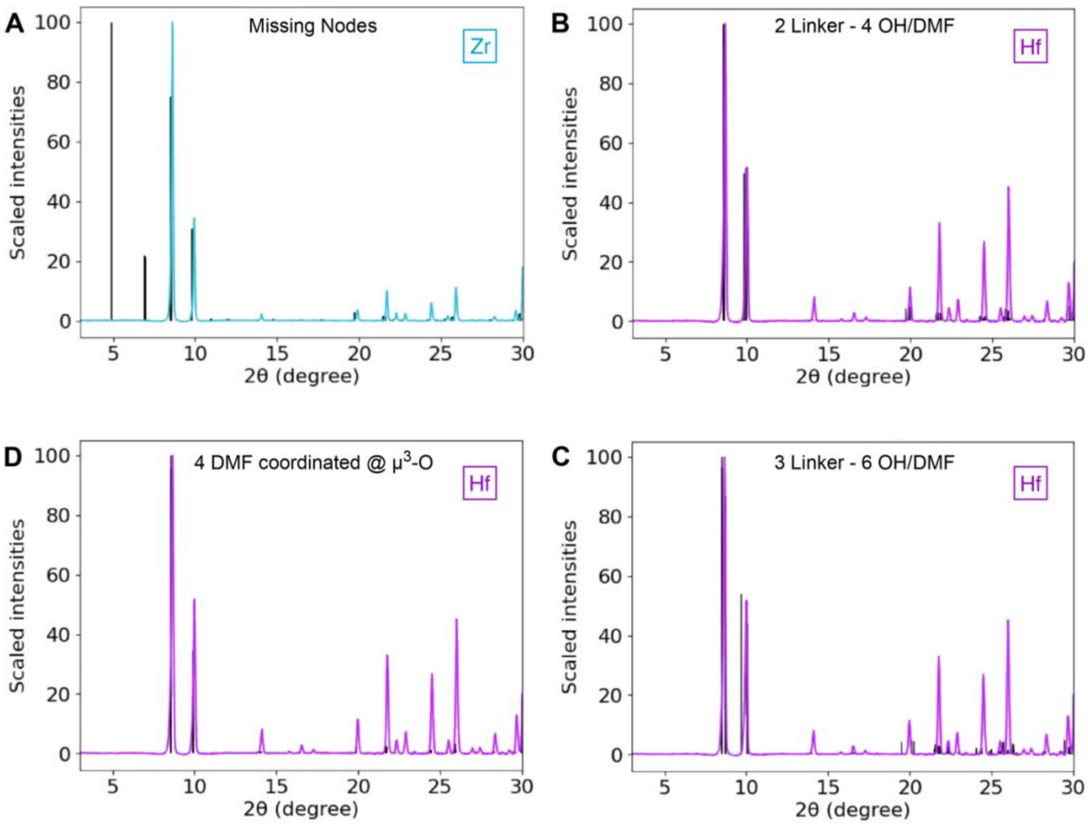

Supplementary Figure S17. Comparison of computational and experimental PXRD patterns for MOF-801(Zr) and MOF-801(Hf). *Computational patterns are shown in black, and experimental data are shown in color.* ***(A)*** *PXRD pattern for MOF-801(Zr) with missing nodes.* ***(B-C)*** *PXRD pattern for MOF-801(Hf) with (B) 2 or (C) 3 missing linkers replaced by 4 or 6 OH/DMF residues, respectively.* ***(D)*** *PXRD pattern for MOF-801(Hf) with 4 DMF molecules coordinated to the* μ^3*-O of the node.*

6.3. Coordination free energies

After getting insights into the MOF framework structure, we next studied the energetics of the interactions of water, guest molecules, and the MOF frameworks. First, we simulated the interaction between water and each potential water-sorption site in the MOFs. Overall, we found that the coordination free energies for the same chemical groups in both Zr- and Hf-based MOF-801 are nearly identical. This supports the hypothesis that the differences in water uptake are due to varying amounts of defects and guest molecules in the material, rather than incorporation of hafnium.

To replicate both the pristine and defective structure, in particular, we used a cluster model at the DFT level (TPSSh-D3BJ/Def2-TZVP) and substituted one linker molecule with DMF/OH- or H_2O/OH- in the defective structure. In both pristine MOF-801 and MOF-808 (Zr or Hf), the primary water binding site is the OH group on the node, which exhibits a coordination free energy of approximately -3.0 kcal/mol in both systems (-1 kcal/mol in the adamantane-like pore). Other coordination sites, such as the O of the carboxylate (COO) group of the linker, the μ^3-O of the node, and the CH group of the linker, exhibit endergonic coordination free energies ranging between 1 and 6 kcal/mol (**Supplementary Table S10**).

Table S10. Coordination free energies according to TPSSh-D3BJ/Def2-TZVP level (T=298 K). *Standard Free Energies in kcal/mol are determined for water and DMF coordination and linker swapping. Abbreviations: adam. = adamantane pore; tetra. = tetrahedral pore*

**Sample**	**MOF-801(Hf)**	**MOF-801(Zr)**	**MOF-808(Hf)**	**MOF-808(Zr)**
**Water Coordination**				
Node-OH	-2.7	-3.1	-1.2 adam.<br>-3.3 tetra.	-1.1 adam.<br>-3.4 tetra.
Linker-CH	1.4	0.9	0.0	-1.9
Formate	N/A	N/A	3.9	2.9
Node-O	2.2	2.5	5.9	5.6
Open metal site	-32.5	-30.4	-33.5	-33.2
**Solvent Replacement in Defective Structures**				
MOF + H_2O/OH- → MOF(def) + linker	-60.3	-61.2	-49.4	-49.5
MOF + DMF/OH- → MOF(def) + linker	-49.4	-50.4	-40.2	-37.2
**DMF Coordination**				
Node-OH	-3.9	-5.5	-3.1 adam.<br>-6.2 tetra.	-3.1 adam.<br>-6.8 tetra.
Open metal site	-22.1	-20.1	-24.2	-20.9

Next, we evaluated the competitive coordination of DMF to the OH group of the node, which serves as the primary water binding site. Our results show that DMF binds more strongly than water in both MOF-801 variants. Specifically, the free energy of DMF binding is - 3.9 kcal/mol in MOF-801(Hf) and - 5.5 kcal/mol in MOF-801(Zr). A similar trend is observed in MOF-808, where the binding of DMF to such OH group in the tetrahedral pore is - 6.2 kcal/mol in MOF-808(Hf) and -6.8 kcal/mol on MOF-808(Zr). Additionally, MOF-808 has a smaller pore, and in both Hf and Zr variants, the coordination free energy of DMF to the OH group in this pore is -3.1 kcal/mol (**Supplementary Table S10**)

Finally, we examined defective structures, where either DMF or water can coordinate to the metal center of the node, replacing the linker. The coordination free energies in both cases are exergonic: -22.1 kcal/mol for Hf-DMF versus -32.5 kcal/mol for Hf-H_2O, and -20.1 kcal/mol for Zr-DMF versus -30.4 kcal/mol for Zr-H_2O; similar values are found in the case of MOF-808 with -24.2 kcal/mol for Hf-DMF versus -33.5 kcal/mol for Hf-H_2O, and -20.9 kcal/mol for Zr-DMF versus -33.2 kcal/mol for Zr-H_2O. While water binds less stable to open metal sites than DMF, Hf binds slightly stronger than Zr by approximately 2 kcal/mol, although this difference falls within the error margin of the DFT calculation. Additionally, the presence of an intermolecular hydrogen bond between water and the OH group results in a more negative overall relative coordination free energy. However, since this hydrogen bond is present in both nodes, the values can be directly compared.

To investigate the coordination energetics of water and DMF with the MOF scaffold further, we performed additional calculations using another functional, B3LYP . **Supplementary Table S11** summarizes the standard free energies of water and DMF coordination, as well as linker swapping calculated at the B3LYP-D3BJ/Def2-TZVP level (T=298 K). The results are in excellent agreement with those obtained using the TPSSh functional, with deviations of about 1-2 kcal/mol, which is within the typical error range of DFT calculations.

Table S11. Coordination free energies according to B3LYP-D3BJ/Def2-TZVP level (T=298 K). *Standard Free Energies in kcal/mol are determined for water and DMF coordination and linker swapping. Abbreviations: adam. = adamantane pore; tetra. = tetrahedral pore*

**Sample**	**MOF-801(Hf)**	**MOF-801(Zr)**	**MOF-808(Hf)**	**MOF-808(Zr)**
**Water Coordination**				
Node-OH	-3.7	-4.0	-1.9 adam.<br>-4.5 tetra.	-1.9 adam.<br>-4.7 tetra.
Linker-CH	0.6	0.0	-1.2	-1.3
Formate	N/A	N/A	2.9	1.8
Node-O	1.2	1.5	4.8	4.5
Open metal site	-32.3	-30.3	-33.5	-33.2
**Solvent Replacement in Defective Structures**				
MOF + H_2O/OH- → MOF(def) + linker	-60.0	-60.5	-49.9	-50.0
MOF + DMF/OH- → MOF(def) + linker	-50.1	-51.1	-41.2	-38.5
**DMF Coordination**				
Node-OH	-5.8	-7.1	-3.1 adam<br>-6.2 tetra	-4.6 adam<br>-9.1 tetra
Open metal site	-23.0	-21.4	-24.6	-21.8

6.4. Methods section: DFT calculations

Periodic density functional theory (DFT) calculations were performed using the Vienna Ab Initio Simulation Package (VASP 6.4.3).[7-8] The Perdew-Burke-Ernzerhof (PBE) exchange-correlation density functional was employed, along with Grimme's D3 dispersion correction with Becke-Johnson damping (D3BJ).[9] Geometry optimizations were carried out using a plane-wave basis set with a kinetic energy cut-off of 520 eV, and standard PAW pseudopotentials were applied for all atoms.[10-11] Convergence criteria for energy and forces were set to 10^{-6} eV and 0.01 eV $Å^{-1}$, respectively, for all optimizations. Given the relatively large size of the unit cell, a 1 × 1 × 1 Γ-centered k-point grid was used in all calculations. After periodic geometry optimization, the software Zeo++ was used to calculate the probe-occupiable, accessible volume (AV) and the surface area.[12] Importantly, PBE-D3BJ was only used for periodic structural optimization of the pristine and defective MOFs. These calculations served exclusively to determine geometric properties (pore volume, accessible volume, and surface area). No adsorption energies, water–framework interactions, or DMF coordination energetics were obtained from PBE calculations, since these are known to be unreliable for hydrogen-bonded systems and their coordination energetics.[13]

Molecular calculations were performed using Kohn Sham density functional theory (KS-DFT) with Gaussian 16 rev B01.[14] Geometry optimization and frequency calculations were performed in the gas phase using the hybrid functional B3LYP, including the empirical dispersion correction with Becke and Johnson damping (D3BJ) and the Ahlrichs def2-SVP basis set for all atoms.[15] Geometry optimization was performed on cluster models extracted from optimized pristine structures done as periodic calculations. To such cluster structures, constraints were applied on the atoms belonging to the original framework, while the additional atoms added to simulate water adsorption, the presence of DMF, and linker defects were optimized without constraints. All energetics relevant to coordination, hydrogen bonding, or defect formation were computed using hybrid functionals. Electronic energies were calculated by single point calculations, including the dispersion correction (D3BJ), at both the B3LYP and TPSSh functionals, with the def2-TZVP basis set for all atoms.[16-17] Vibrational frequency calculation at 298 K was performed at the optimization level of theory. The free energy correction applied to the electronic energy was calculated based on the frequency calculation, setting a cut-off for small vibrations to 100 cm^{-1}.[18] Thus, all coordination free energies and mechanistic interpretations presented in this work rely exclusively on hybrid-functional (B3LYP/TPSSh) energetics, while PBE-D3BJ was used solely to obtain periodic structural models for porosity analysis.

Supplementary Note 7. Solvent Blocking: Controlling Residual DMF in MOF-801(Zr)

To assess the influence of residual dimethylformamide (DMF) confined within the pores of MOF-801(Zr) on its water adsorption behavior, we prepared a series of samples with systematically varied DMF contents. Residual DMF is known to occupy pore volume and thereby reduce the number of accessible adsorption sites for water molecules. To control the amount of DMF remaining in the framework, we employed a set of carefully designed solvent-exchange steps that enabled us to incrementally adjust the residual DMF concentration in the activated materials.

7.1 Synthesis and Washing Protocol

Seven independent batches of MOF-801(Zr) were synthesized following the procedure described in **Supplementary Note 2**. After completion of the synthesis, the reaction mixtures were transferred into 50 mL Falcon tubes, and the MOF crystals were separated from the DMF mother liquor by centrifugation (Hettich EBA 20, 5000 rpm) for 10 min. The supernatant was discarded, and the as-synthesized, unwashed MOF solids were redispersed in 20 mL of methanol. The resulting methanol/MOF suspensions were subjected to solvent exchange by gentle rotary mixing for 8 h. Subsequently, the suspensions were centrifuged again, the methanol supernatant was removed, and fresh methanol was added. To obtain samples with systematically varied amount of residual DMF, this solvent exchange cycle was repeated 0, 1, 2 (2x), 3, 4, and 7 times for Batches 1–7, respectively. After the final washing step, all samples were activated at 80 °C for 24 h prior to further characterization.

7.2 Bulk Characterization and Quantification of DMF

All MOF-801(Zr) batches prepared with different DMF-washing protocols were characterized using a combination of structural, morphological, and spectroscopic techniques to assess framework integrity and to quantitatively determine residual solvent content. The employed methods included SEM, PXRD, and NMR spectroscopy.

7.2.1. Structural characterization

SEM images (**Supplementary Figure S18**) were acquired using a field-emission scanning electron microscope (FEI Helios G3 UC) equipped with an energy-dispersive X-ray spectroscopy (EDX) system (X-Max 80, Oxford Instruments). Imaging was performed at an accelerating voltage of 2 kV. Prior to SEM analysis, all samples were coated with a 7.5 nm carbon layer to enhance surface conductivity and minimize charging effects. SEM analysis confirms that the DMF washing protocol does not affect particle morphology, size distribution, or crystal integrity of MOF-801(Zr). Variations in solvent content and water uptake therefore do not originate from changes in particle morphology or size.

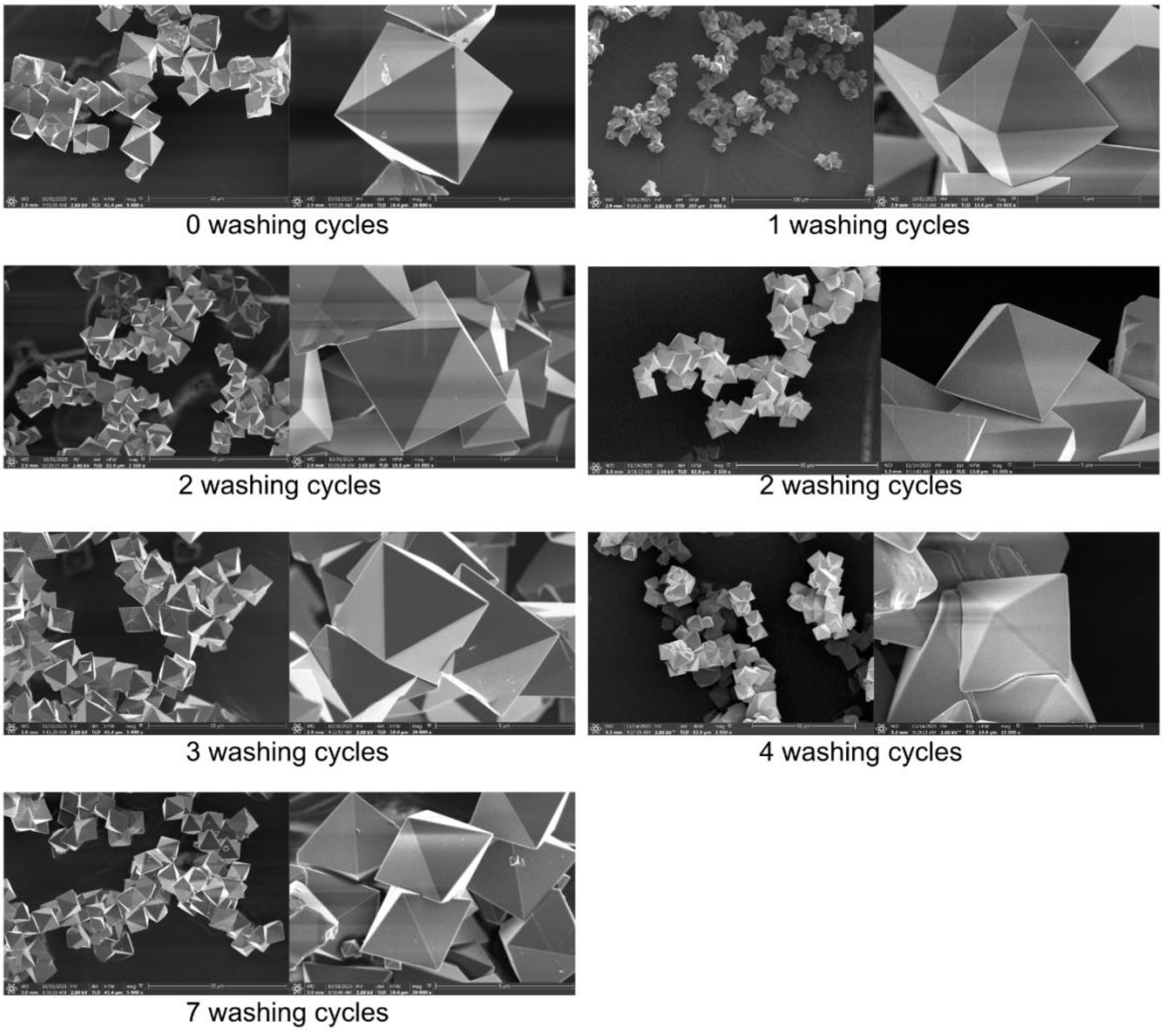

Supplementary Figure S18. SEM images of MOF-801(Zr) samples subjected to different numbers of washing steps. *Representative scanning electron microscopy (SEM) images of MOF-801(Zr) particles obtained from batches after an increasing number of washing cycles (Batch 1–7, as indicated) to remove DMF. No changes in particle morphology, size distribution and crystal integrity are observed*

PXRD patterns (**Supplementary Figure S19**) were recorded on a STOE Stadi P diffractometer (STOE & Cie. GmbH, Darmstadt) using Cu Kα radiation (λ = 1.54060 Å) at an acceleration voltage of 40 kV and a tube current of 40 mA. All diffraction patterns were normalized to the reflection at 2θ = 8.5° to facilitate direct comparison between samples. The PXRD data confirm that varying the number of DMF-washing steps does not alter the phase purity or long-range crystallinity of MOF-801(Zr). Thus, differences in water uptake and adsorption behavior arise from pore-level effects, rather than from structural degradation or framework collapse.

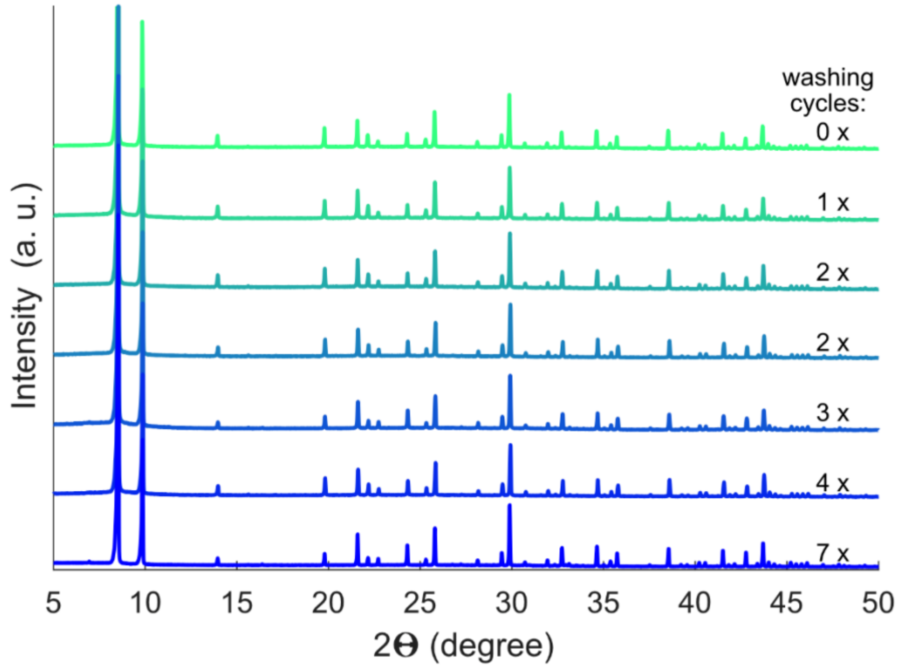

Supplementary Figure S19. Corresponding Powder X-ray diffraction (PXRD) patterns of MOF-801(Zr) recorded for samples exposed to an increasing number of DMF washing cycles. *From top to bottom: Batch 1 (0 washing cycles; green), Batch 2 (1 washing cycle), Batch 3 (2 washing cycles), Batch 4 (2 washing cycles), Batch 5 (3 washing cycles), Batch 6 (4 washing cycles), and Batch 7 (7 washing cycles; dark blue). All diffraction patterns are normalized to the reflection at 2θ = 8.5°.*

7.2.2. Data evaluation and quantification of residual DMF by NMR

Quantitative analysis of residual dimethylformamide (DMF) and formic acid (FA) was carried out based on the ^{1}H NMR spectra (**Supplementary Figure S20**). Sample digestion, NMR data acquisition, and spectral processing followed the procedures described in **Supplementary Note 5** and **1.4**, respectively.

Table S12. Quantification of residual DMF and formic acid in MOF-801(Zr) samples derived from ^{1}H NMR spectra. *Number of DMF and FA molecules per fumaric acid linker, calculated from the integrated ^{1}H NMR resonances shown in* ***Supplementary Figure S20:*** *DMF at 2.70, 2.86, and 7.58 ppm; FA at 8.32 ppm. Values are reported for MOF-801(Zr) samples subjected to increasing numbers of washing cycles (Batch 1–7), illustrating the progressive removal of residual DMF with increasing washing intensity while retaining FA-related species.*

**Sample**	**DMF / Linker (2.70 ppm)**	**DMF / Linker (2.86 ppm)**	**DMF / Linker (7.58 ppm)**	**FA / Linker (8.32 ppm)**
**Batch 1; 0 washing cycles**	0.6466	0.6474	0.7041	0.3857
**Batch 2; 1 washing cycles**	0.6224	0.6364	0.6256	0.4052
**Batch 3; 2 washing cycles**	0.6337	0.6492	0.6567	0.4178
**Batch 4; 2 washing cycles**	0.3170	0.3170	0.3293	0.3768
**Batch 5; 3 washing cycles**	0.0285	0.0294	0.0442	0.1410
**Batch 6; 4 washing cycles**	0.3683	0.3798	0.3814	0.3317
**Batch 7; 7 washing cycles**	0.0021	0.0027	0.0001	0.1422

The ratios of DMF and FA molecules relative to the fumaric acid linker were determined by integrating the characteristic proton resonances. DMF-to-linker ratios were extracted from the methyl resonances at 2.70 and 2.86 ppm and the formyl proton at 7.58 ppm, while the FA-to-linker ratio was obtained from the resonance at 8.32 ppm. The fumaric acid linker signal at 6.3 ppm served as an internal reference for normalization. The quantitative results for all batches are summarized in **Supplementary Table S12**.

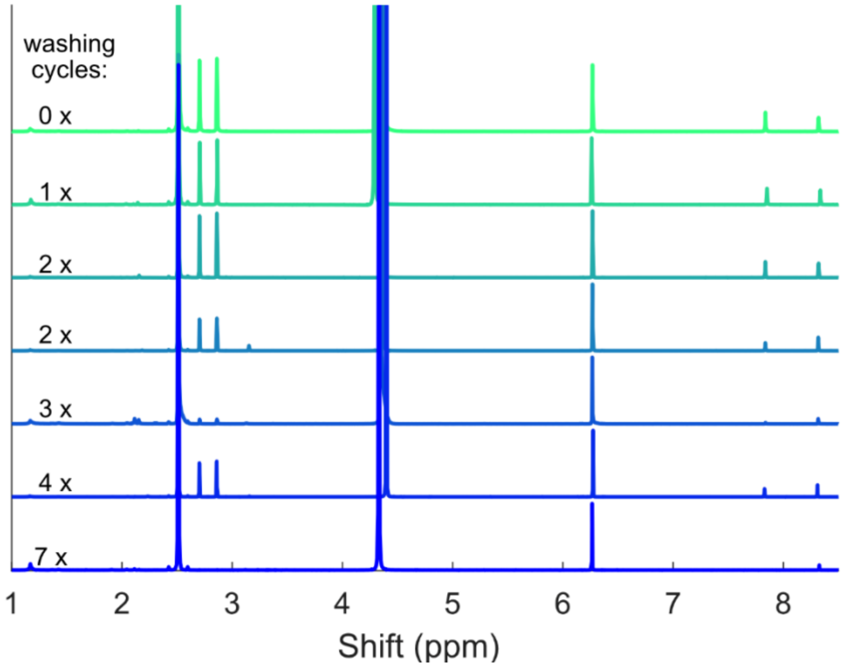

Supplementary Figure S20. ***^{1}H NMR spectra of MOF-801(Zr) samples subjected to increasing number of washing steps.*** *^{1}H NMR spectra of MOF-801(Zr) recorded for samples exposed to an increasing number of DMF washing cycles. From top to bottom: Batch 1 (0 washing cycles; green), Batch 2 (1 washing cycle), Batch 3 (2 washing cycles), Batch 4 (2 washing cycles), Batch 5 (3 washing cycles), Batch 6 (4 washing cycles), and Batch 7 (7 washing cycles; dark blue). Signals associated with residual DMF (N–CH_3 resonances at ~2.7–3.1 ppm) decrease progressively with increasing washing intensity, while the fumaric acid linker resonance at 6.3 ppm remains constant. All spectra are normalized to the fumaric acid linker peak at 6.3 ppm.*

The quantified DMF-to-linker ratios show an overall decrease with increasing numbers of washing cycles, confirming the effective removal of pore-confined DMF through solvent exchange. However, several batches deviate from a monotonic trend—for example, the sample washed three times exhibits an unexpectedly low DMF content. As a result, the reduction of residual DMF does not follow a simple linear or exponential dependence on the number of washing steps. This variability demonstrates that the residual DMF content cannot be reliably inferred solely from the number of washing cycles. Therefore, the residual DMF content must be experimentally determined for each batch individually. This quantitative NMR-based assessment is therefore essential for correlating solvent content with water uptake behavior and for ensuring reproducibility across independently prepared MOF-801(Zr) samples.

7.3 Quantification of residual DMF using vibrational spectroscopy

Raman microscopy was additionally used to probe characteristic vibrational modes of residual DMF and formate species. While Raman microscopy was initially intended as a complementary method to NMR, it became evident that a single-crystal evaluation of MOF-801(Zr) is essential to stablish a direct link between the water uptake behavior and DMF content. Bulk-averaged techniques such as NMR provide reliable ensemble-averaged solvent contents, but cannot resolve particle-to-particle variations in residual DMF loading. Such heterogeneity is expected due to differences in crystal size, morphology, and pore accessibility following the washing steps. In contrast, Raman microscopy enables spatially resolved analysis and is therefore uniquely suited to probe residual solvent content on individual MOF crystals. All spectra were recorded using identical acquisition settings as used for Raman-based water adsorption measurements (see **Supplementary Note 1.4**)

7.3.1 Raman Spectra of chemicals used during synthesis

To reliably use Raman spectroscopy for quantification of residual DMF, we first identified and assigned the vibrational signatures of all molecular species involved in synthesis and washing. Establishing these reference spectra allows for disentangling overlapping contributions in the C–H stretching region and identifying spectral features suitable as robust internal references.

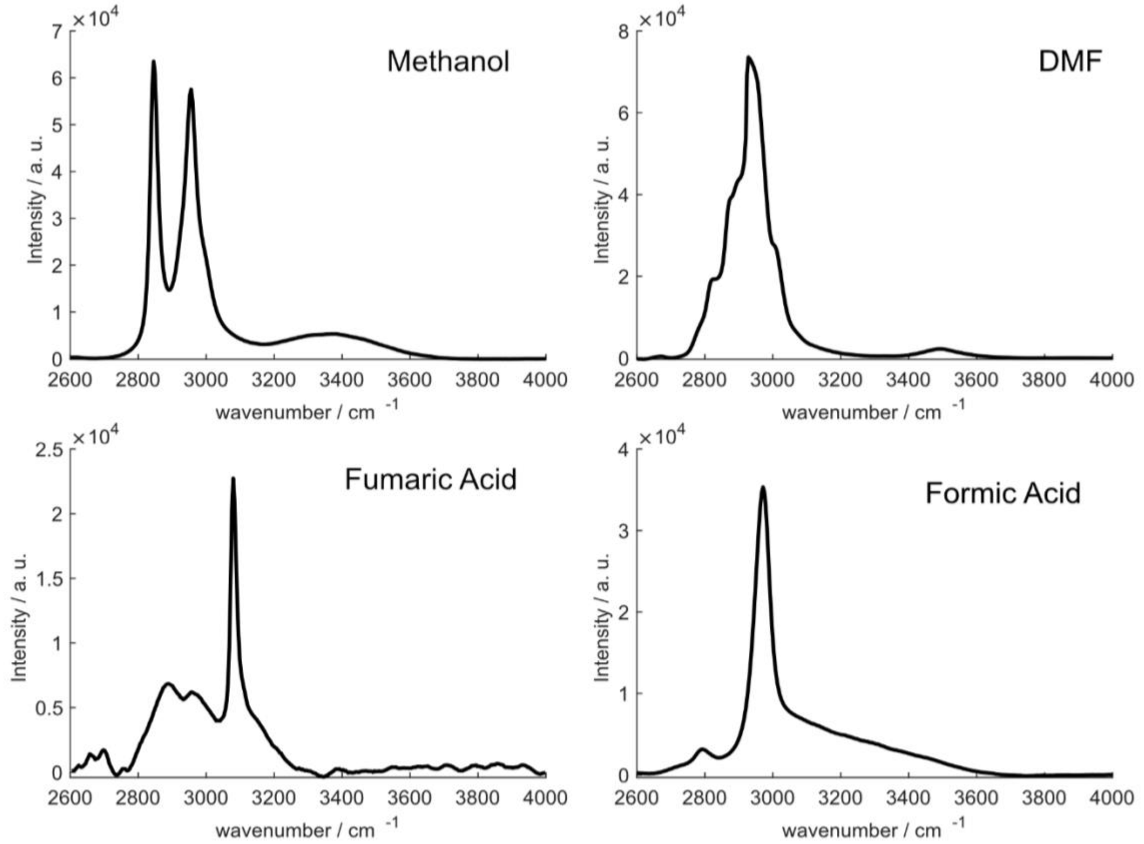

Supplementary Figure S21. Vibrational signatures of molecular species involved in the synthesis of MOF-801(Zr). *Raman spectra recorded in the spectral range between 2600 and 4000 cm^{-1} for **(A)** methanol, **(B)** DMF, **(C)** fumaric acid, and **(D)** formic acid. The spectra highlight characteristic vibrational features associated with C–H and O–H stretching modes, enabling spectral discrimination of the individual molecular species based on their distinct vibrational fingerprints.*

Supplementary Figure S21 shows the Raman spectra of all molecular species involved in the synthesis and washing of MOF-801(Zr), including methanol, DMF, fumaric acid, and formic acid. All molecular species exhibit Raman-active modes in the C–H stretching region between 2800 and 3000 cm^{-1}, resulting in significant spectral overlap. This overlap complicates independent quantification of individual components based solely on this spectral region. In contrast, fumaric acid—the organic linker of MOF-801(Zr)—shows a distinct and intense C–H stretching vibration around 3100 cm^{-1} that is not shared by the solvent molecules. This band therefore provides a robust internal reference that is insensitive to residual solvent content.

7.3.2 Raman of batches after different numbers of washing cycles

Supplementary Figure S22 shows representative Raman spectra acquired from three individual MOF-801(Zr) crystals of the most extensively washed batch (left) and unwashed batch (right). All crystals were dried under a continuous N_2 flow prior to measurement to minimize contributions from physisorbed water. Two strong Raman bands are consistently observed at approximately 3100 cm^{-1} and 3700 cm^{-1}, assigned to the C–H stretching vibration of the fumaric acid linker and to the μ_3–OH stretching vibration of the Zr_6-oxo cluster in MOF-801(Zr), respectively. Spectral features between 2800 cm^{-1} and 2950 cm^{-1} arise from a superposition of aliphatic C–H stretching modes of residual species originating from synthesis and washing—namely DMF, methanol, and formic acid—together with contributions from the linker molecule. Reference Raman spectra of these species are provided in **Supplementary Figure S21**.

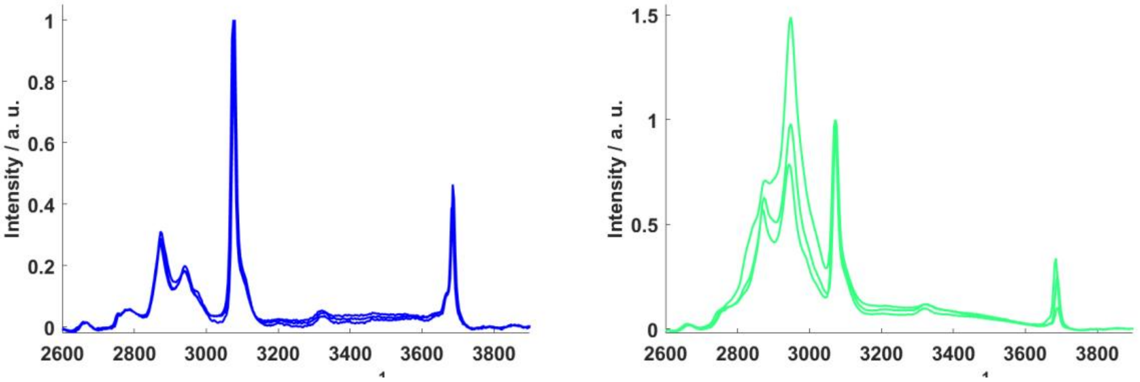

Supplementary Figure S22. Raman spectra of MOF-801 (Zr) for the most washed and the unwashed batches. Left: *Raman spectra of three individual crystals from Batch 7, washed seven times with methanol.* ***Right:*** *Raman spectra of three individual crystals from Batch 1, activated directly after synthesis without prior washing. All spectra are background-corrected and normalized to the C–H stretching vibration of the fumaric acid linker.*

Consistent with the NMR measurements, the Raman spectra exhibit a pronounced change in both intensity and spectral shape of the band at about 2950 cm^{-1} upon washing. Based on its characteristic line shape and comparison with the reference spectra, this band is dominated by the C–H stretching vibrations of residual DMF. The quantitative NMR results (**Supplementary Table S12**) show that (1) methanol is essentially absent and that (2) formic acid is present at significantly lower concentrations than DMF in all batches. For these reasons, the DMF-dominated band near 2950 cm^{-1} was fitted with two pseudo-Voigt components (red and pink curves), to account for the small shoulder around 3000 cm^{-1} in the DMF spectrum

(**Supplementary Figure S23),** while the fumaric acid linker peak at 3100 cm^{-1} was approximated only via a single pseudo-Voigt function. Consequently, the ratio between the integrated area of this DMF-dominated band and the area of the fumaric acid linker C–H stretching vibration at 3100 cm^{-1} was used as a quantitative measure for residual DMF content at the single-crystal level.

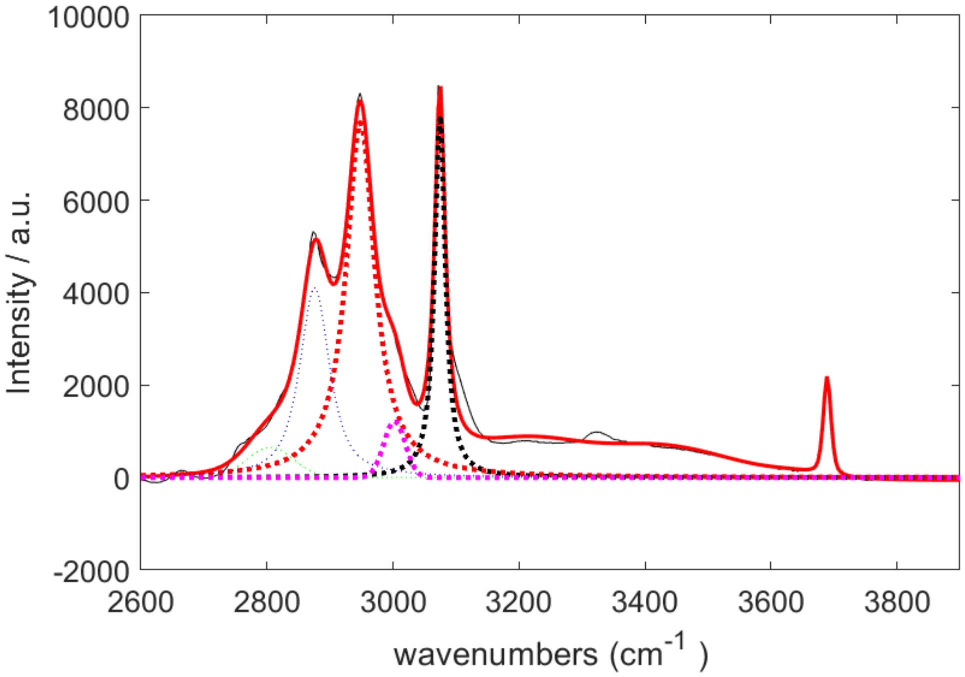

Supplementary Figure S23. Raman spectrum of an unwashed MOF-801(Zr) single crystal illustrating the fitting procedure used for quantitative analysis of residual DMF. *The C–H stretching vibration of the fumaric acid linker at ~3100 cm^{-1} is fitted with a single pseudo-Voigt function, while the DMF-dominated band around ~2950 cm^{-1} is fitted using two pseudo-Voigt components to account for spectral asymmetry and a minor high-wavenumber shoulder. The extracted integrated peak areas are used to determine the DMF-to-linker ratio.*

To relate the Raman-based DMF-to-linker area ratios to absolute DMF concentrations, the ratios obtained from Raman spectroscopy were plotted against the number of DMF molecules per linker molecule determined from ^{1}H NMR for each batch (**Supplementary Figure S24**). The NMR-derived values represent ensemble averages over millions of crystals, whereas the Raman-derived ratios reflect distributions obtained from individual single crystals. Substantial variations in residual DMF content are observed between different crystals within the same batch, highlighting pronounced particle-to-particle heterogeneity—and thus the necessity of single-crystal analysis.

Despite this heterogeneity, the batch-averaged Raman ratios follow a linear trend with the NMR-derived DMF-to-linker ratios, consistent with both techniques scaling linearly with concentration. The non-zero y-axis offset of the linear fit is attributed to residual contributions from other synthesis-related guest molecules (formic acid about 10% of both fractions), as well as a minor contribution from the fumaric acid linker itself in the spectral region used for DMF quantification. During fitting, the y-axis offset was constrained to a maximum value of 0.48—the lowest experimentally observed Raman DMF-to-linker area ratio—to avoid non-physical negative DMF concentrations upon back-calculation. By inverting the linear fit, a calibration

function was obtained that allows conversion of Raman-derived intensity ratios into absolute numbers of DMF molecules per linker molecule and a quantitative determination of residual DMF content at the single-crystal level using Raman spectroscopy.

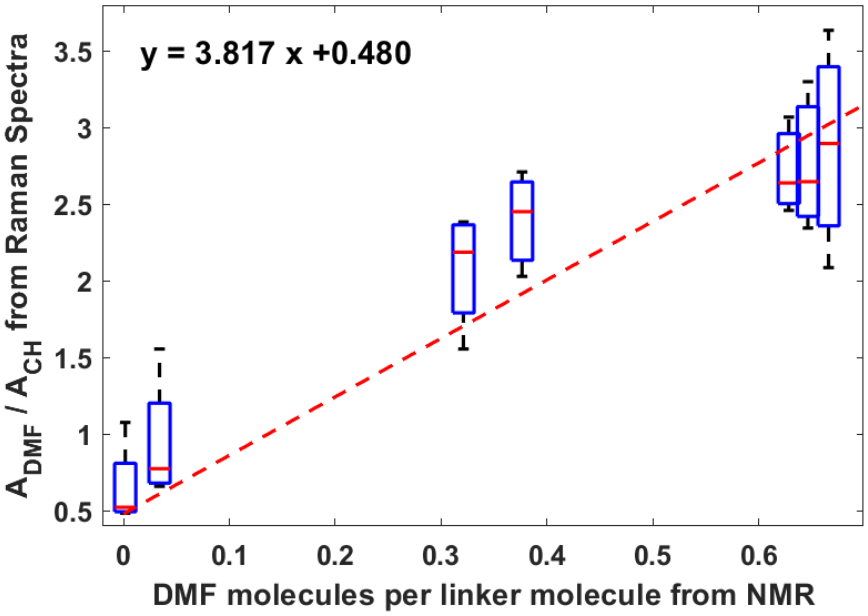

Supplementary Figure S24. Correlation between Raman-derived DMF intensity ratios and NMR-quantified DMF content. *Ratio of the integrated area of the DMF-dominated C–H stretching band (~2950 cm⁻¹) to the fumaric acid linker C–H stretching band (~3100 cm⁻¹) obtained from Raman spectroscopy, plotted as a function of the number of DMF molecules per linker molecule determined from ^{1}H NMR. Box plots represent distributions obtained from three individual single crystals per batch. The dashed line shows a linear fit through the batch-averaged Raman ratios. The y-axis offset of the fit was constrained to a maximum value of 0.48, corresponding to the lowest experimentally observed DMF-to-linker area ratio, to avoid non-physical negative DMF contents upon back-calculation.*

Supplementary Note 8. Kinetic measurements

This supplementary note addresses an unexpected issue encountered during kinetic measurements with emCCD cameras, commonly used for data acquisition in *in-situ* Raman spectroscopy. When acquiring water sorption kinetics in MOFs, we observed that longer integration times led to artificially slower kinetics, especially when the integration time exceeded the readout time by several orders of magnitude. This discrepancy was traced to the missing ***frame transfer mode*** in the software during data acquisition, even though unsynchronized frame transfer should occur on the millisecond time scale – three orders of magnitudes smaller than the frame time. While the exact electronic cause remains unclear from the supplier's side, the implications are significant: without frame transfer mode, the true dynamics of transport systems can be misrepresented on the second scale. As the integration time from one acquisition influences the next, actual water uptake capacities are underestimated. While activating frame transfer mode resolves this issue, we strongly recommend verifying the correct functioning of the data acquisition when using emCCD cameras. The following note provides a detailed analysis of this issue, including experimental tests with a silicon wafer and known excitation kinetics to demonstrate how activating frame transfer successfully resolves the discrepancy.

8.1. Readout of CCD Cameras

During illumination of the Charge Coupled Device (CCD) chip, electrons in the pixels are excited from the valence to the conduction band. By applying a positive voltage to the gate electrode at each pixel, these electrons are trapped in potential wells at their respective pixels (**Supplementary Figure S25A**). The number of trapped electrons is proportional to the intensity of incident light at that pixel. For readout, all rows on the chip are first shifted by one row towards the readout row (**Supplementary Figure S25B**). The pixels in the readout row (blue) are then transferred one by one to an analog-to-digital converter for processing (**Supplementary Figure S25C**). Once the readout row is empty, the next row is shifted, and the process repeats until all pixels in the row are read and the next line is transferred (**Supplementary Figure S25D**). During the readout process, the chip must be shielded from illumination to prevent the excitation of new electrons, which would lead to signals being detected at the wrong pixel. However, this blocking should not be necessary if the integration/illumination time for each frame is significantly longer than the readout time, as is the case for spontaneous Raman measurements. Generally, the readout time depends on the chip size, camera type, and desired signal-to-noise ratio. Modern CCD cameras with 512 by 512 pixels achieve readout rates of around 50 frames per second (fps), limiting time resolution to about 20 ms. The time resolution can be improved by transferring all the trapped electrons to a second, dark readout chip. This allows the chip to be illuminated again as soon as the electrons are transferred, while the dark chip is read out pixel by pixel (**Supplementary Figure S25D-G**) on the sub 10 ms time scale.

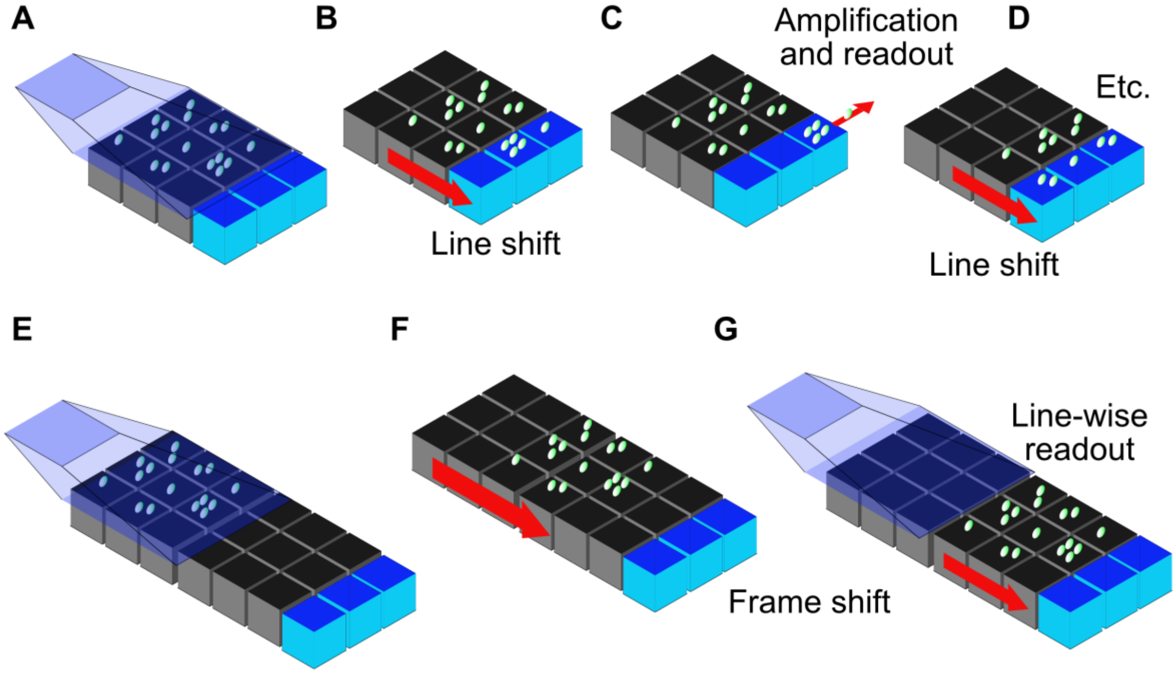

Supplementary Figure S25. Readout process of emCCD cameras. (A) *Electrons are excited from the valence to the conduction band and trapped at each pixel of the chip during illumination.* ***(B-D)*** *For readout, rows are shifted one by one towards the readout row, and pixels are processed sequentially by an analog-to-digital converter.* ***(E-G)*** *Faster time resolution is achieved by transferring trapped electrons to a secondary dark chip (frame transfer mode), allowing for simultaneous illumination and line-wise readout of the shadowed area in the meantime.*

8.2. Measurement of kinetic series with long-term exposure

The adsorption and desorption kinetics previously reported appeared on a slower timescale than observed in this study.[1] In both investigations, all Raman spectra were collected using the same detection system based on a Kymera-328i Spectrometer (Andor Solis) equipped with an emCCD camera (Andor; iXon Ultra 897). The acquisition settings and the camera were controlled by the camera supplier's software *Andor Solis for Imaging V4.30* (Oxford Instruments). To our (unpleasant) surprise, using this software in *kinetic mode*, the measured speed of the process depends on the integration time of each spectrum if *"frame transfer mode"* is not activated during the measurement. While physically, longer integration times reduce time resolution, the observed slowdown in kinetics cannot be fully attributed to this factor alone. This effect is most pronounced when the integration time is much longer than the readout time, where the lack of frame transfer should have minimal influence on the observed kinetics.

To address this issue, we conducted a Raman experiment with signature changes at known kinetics to test the setup's performance. Specifically, we measured the intensity of the Raman peak of a silicon wafer at 520 cm^{-1} while modulating the laser power at a controlled rate. The laser power was varied using a fixed polarizer and a motorized $\lambda/2$ plate. The rotation of the $\lambda/2$ plate was precisely controlled by the supplier's software (Thorlabs; Kinesis software) and confirmed via a photodiode. Initially, the laser was blocked by rotating the polarizer to a perpendicular position. Then, the laser power was gradually increased at a known rate by rotating the $\lambda/2$ plate. The evolution of the silicon peak was recorded in *kinetic mode*, without frame transfer, at different integration times. As shown in **Supplementary Figure S26**, the same process appears to be slower at longer integration times. According to the theoretical model, the laser power should reach its maximum after 14 seconds, based on the set rotation speed of the motorized $\lambda/2$ plate. However, at integration times of 10 and 20 seconds, saturation was not observed, even at data points taken more than one full integration time after saturation should have been reached. This suggests that the integration interval is affected by the previous data acquisition, leading to inaccurate measurements. This can be fixed by activating frame transfer. Performing the same experiment with activated frame transfer, the different integration times yield the same kinetic behavior, as it would be expected when measuring the same process. Also, different speeds of the $\lambda/2$ plate can be easily distinguished.

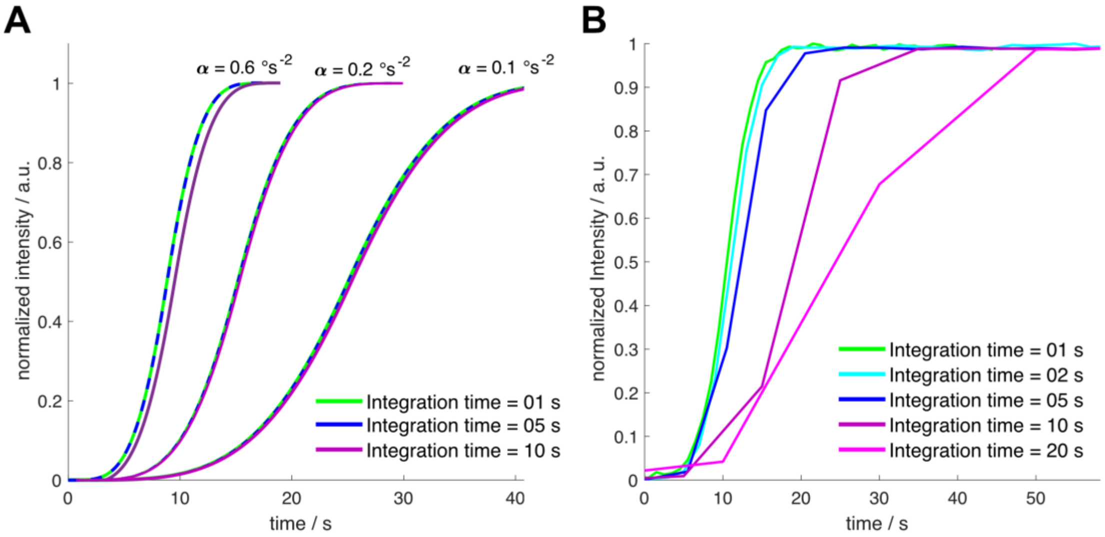

Supplementary Figure S26. Effect of integration time on measured kinetics. *The intensity of the silicon Raman peak at 520 cm*$^{-1}$ *was recorded as the laser power was gradually increased at a known rate using a motorized λ/2 plate.* ***(A)*** *Normalized measured Raman signature at three different angular accelerations of the λ/2 plate (α = 0.6 °s*$^{-2}$*, 0.2 °s*$^{-2}$*, and 0.1 °s*$^{-2}$*), each measured at three different integration times (1 s, 5 s, and 10 s) with frame transfer activated.* ***(B)*** *Measured Raman signature at varying integration times without Frame transfer activated at an angular acceleration of the λ/2 plate of 0.5 °s*$^{-2}$*.*

8.3. Comparison of SC kinetics for Zr- and Hf- based MOF-801 and MOF-808

Achieving fast and reversible switching between adsorption and desorption is crucial for practical applications of adsorbents used for water harvesting. The efficiency of this process depends on the kinetics of uptake and release, as well as the cycling stability. To investigate the adsorption (and desorption) kinetics, we exposed (initially dry) MOF particles alternatingly to an N_2 stream first and afterward humid air (or vice versa) while monitoring their response using Raman spectroscopy. To start with MOF crystals in a dry state at t_0 = 0 s, we flushed them with N_2 for 30 minutes first before exposing them to humid air (RH ~ 40%). We tracked changes in the OH-stretch region over time, observing a rapid increase in the water band from ~3000 to 3800 cm^{-1} until saturation. Both MOF-801 and MOF-808 crystals reached saturation in less than 10 seconds, with MOF-801 saturating in just 6 seconds (**Supplementary Figure S27A**). This observation aligns well with prior studies.[1] The crystals, with diameters of around 4-5 µm (**Supplementary Figure S27; Supplementary Note 3.1**), display diffusion times on the micro- to millisecond time scale. Notably, substituting zirconium with hafnium did not significantly alter the saturation kinetics.

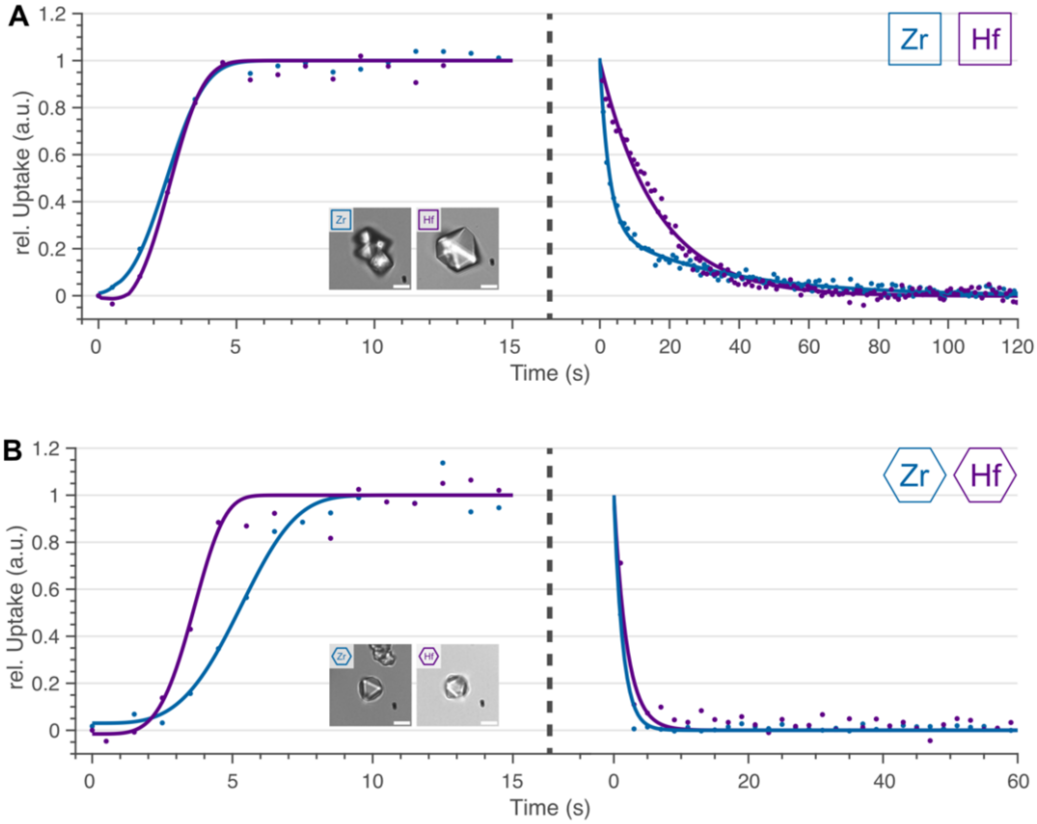

Supplementary Figure S27. Kinetics of water uptake and water release. *Kinetic curves for water adsorption (left) and desorption (right) for **(A)** MOF-801 and **(B)** MOF-808 at ~40% and 0% RH based on (blue) zirconium and (purple) hafnium. Adsorption kinetics were measured at a time resolution of 0.5 s. Desorption Kinetics were measured at a time resolution of 1 s for MOF-801 (Zr/Hf) and 2s for MOF-808(Zr/Hf). Insets: Images of MOF-801 and MOF-808 single crystals taken by bright field. Scale bar: 5 µm.*

The desorption kinetics were examined using the same methods; however, in this case, the crystals were initially exposed to humid air (RH ~40%) until saturation (for 5 min), and at t_0 = 0 s, they dried using an N_2 stream. The desorption process was monitored by tracking changes in the OH-stretch region. For both MOF-808 samples, water desorbed almost instantaneously upon exposure to nitrogen, reducing the crystals' water content to 10% of their uptake capacity in under 5 seconds. In contrast, desorption in MOF-801 (both Hf- and Zr-based) was delayed and exhibited a biexponential decay. We assign the fast component in the decay to the desorption kinetics of water, while the slow component originates from the time needed to exchange the air and reduce the relative humidity (RH) around the crystal before desorption occurs. This behavior was not observed in MOF-808 samples, where desorption began at significantly higher humidity levels. Despite the delayed desorption, the adsorbed water content in MOF-801(Zr) dropped to 20% of saturation in under 15 seconds and in MOF-801(Hf) in under 30 seconds. Following DFT simulations, we explain the slower desorption in MOF-801(Hf) compared to MOF-801(Zr) by blocking pores with DMF molecules causing slower diffusion within the material.

8.4. Theoretical limits in water harvesting by Zr- and Hf- based MOF-801 and MOF-808

The theoretical efficiency limits for atmospheric water harvesting (AWH) were estimated based on adsorption and desorption times in conjunction with the amount of water adsorbed at saturation. Full uptake capacities were derived from single-crystal isotherms at saturation, while effective capacities accounted for residual water that could not be removed during rapid desorption. Adsorption times were conservatively set to 5 seconds, whereas desorption times were defined as the duration required to release 80% (MOF-801) or 90% (MOF-808) of the adsorbed water. The total cycle time was calculated by summing the adsorption and desorption times, allowing for the determination of the maximum number of cycles per hour. The theoretical efficiency limit was then calculated by multiplying the effective water uptake capacity by the maximum cycle frequency, providing an upper bound for water collection rates in liters per kilogram per hour (L kg^{-1} h^{-1}). All values are summarized in **Supplementary Table S13**.

Table S13. Uptake capacities, cycling speed, and theoretical efficiency limits. *Full capacities are derived from SC isotherms at saturation. However, practical uptake capacities are lower due to residual water in MOFs that cannot be fully removed on fast time scales. Adsorption times are estimated conservatively to 5 s, while given desorption times correspond to the duration needed to remove 80% (MOF-801) or 90% (MOF-808) of adsorbed water, depending on the residual water content. Cycles per hour are determined based on the total time per cycle, comprising both adsorption and desorption times. The theoretical efficiency limit was estimated by multiplying the corrected full capacity by the number of cycles possible per hour.*

**Sample**	**Full Capacity**	**Effective Capacity**	**Adsorption Time (Sat.)**	**Desorption Time (to eff.)**	**Cycles per hour**	**Theoretical efficiency limits**
MOF-801(Zr)	0.35 g g^{-1}	80 %	5 s	13 s	200	56 L $kg^{-1}h^{-1}$
MOF-801(Hf)	0.12 g g^{-1}	80 %	5 s	26 s	116	11 L $kg^{-1}h^{-1}$
MOF-808(Zr)	0.78 g g^{-1}	90 %	5 s	1.7 s	534	375 L $kg^{-1}h^{-1}$
MOF-808(Hf)	0.2 g g^{-1}	90 %	5 s	1.2 s	580	104 L $kg^{-1}h^{-1}$

Supplementary References

[1] A. Fuchs, F. Knechtel, H. Wang, Z. Ji, S. Wuttke, O. M. Yaghi, E. Ploetz, “Water Harvesting at the Single-Crystal Level“, *J. Am. Chem. Soc.* **2023**, *145*, 14324-14334.

[2] H. Furukawa, F. Gandara, Y. B. Zhang, J. Jiang, W. L. Queen, M. R. Hudson, O. M. Yaghi, “Water adsorption in porous metal-organic frameworks and related materials“, *J. Am. Chem. Soc.* **2014**, *136*, 4369-4381.

[3] Y.-M. Gu, Y.-Y. Yuan, S. Qadir, Z.-S. Yuan, S.-S. Zhao, T.-J. Sun, X.-W. Liu, S.-D. Wang, “Mixed-Linker Metal-Organic frameworks for carbon and hydrocarbons capture under moist conditions“, *Chem. Eng. J.* **2022**, *433*, 134447.

[4] Z. Hu, Y. Wang, D. Zhao, “The chemistry and applications of hafnium and cerium(iv) metal–organic frameworks“, *Chem. Soc. Rev.* **2021**, *50*, 4629-4683.

[5] A. Fuchs, P. Mannhardt, P. Hirschle, H. Wang, I. Zaytseva, Z. Ji, O. M. Yaghi, S. Wuttke, E. Ploetz, “Single Crystals Heterogeneity Impacts the Intrinsic and Extrinsic Properties of Metal-Organic Frameworks“, *Adv. Mater.* **2022**, *34*, 2104530.

[6] P. H. M. Andrade, N. Henry, C. Volkringer, T. Loiseau, H. Vezin, M. Hureau, A. Moissette, “Iodine Uptake by Zr-/Hf-Based UiO-66 Materials: The Influence of Metal Substitution on Iodine Evolution“, *ACS Appl. Mater. Interfaces* **2022**, *14*, 29916-29933.

[7] G. Kresse, J. Hafner, “Ab initio molecular dynamics for liquid metals“, *Phys. Rev. B* **1993**, *47*, 558-561.

[8] G. Kresse, J. Furthmüller, “Efficiency of ab-initio total energy calculations for metals and semiconductors using a plane-wave basis set“, *Comput. Mater. Sci.* **1996**, *6*, 15-50.

[9] S. Grimme, S. Ehrlich, L. Goerigk, “Effect of the damping function in dispersion corrected density functional theory“, *J. Comput. Chem.* **2011**, *32*, 1456-1465.

[10] P. E. Blöchl, “Projector augmented-wave method“, *Phys. Rev. B* **1994**, *50*, 17953-17979.

[11] G. Kresse, D. Joubert, “From ultrasoft pseudopotentials to the projector augmented-wave method“, *Phys. Rev. B* **1999**, *59*, 1758-1775.

[12] T. F. Willems, C. H. Rycroft, M. Kazi, J. C. Meza, M. Haranczyk, “Algorithms and tools for high-throughput geometry-based analysis of crystalline porous materials“, *Microporous Mesoporous Mater.* **2012**, *149*, 134-141.

[13] K. Sharkas, K. Wagle, B. Santra, S. Akter, R. R. Zope, T. Baruah, K. A. Jackson, J. P. Perdew, J. E. Peralta, “Self-interaction error overbinds water clusters but cancels in structural energy differences“, *Proc. Natl. Acad. Sci. U.S.A.* **2020**, *117*, 11283-11288.

[14] M. J. Frisch, G. W. Trucks, H. B. Schlegel, G. E. Scuseria, M. A. Robb, J. R. Cheeseman, G. Scalmani, V. Barone, G. A. Petersson, H. Nakatsuji, X. Li, M. Caricato, A. V. Marenich, J. Bloino, B. G. Janesko, R. Gomperts, B. Mennucci, H. P. Hratchian, J. V. Ortiz, A. F. Izmaylov, J. L. Sonnenberg, Williams, F. Ding, F. Lipparini, F. Egidi, J. Goings, B. Peng, A. Petrone, T. Henderson, D. Ranasinghe, V. G. Zakrzewski, J. Gao, N. Rega, G. Zheng, W. Liang, M. Hada, M. Ehara, K. Toyota, R. Fukuda, J. Hasegawa, M. Ishida, T. Nakajima, Y. Honda, O. Kitao, H. Nakai, T. Vreven, K. Throssell, J. A. Montgomery Jr., J. E. Peralta, F. Ogliaro, M. J. Bearpark, J. J. Heyd, E. N. Brothers, K. N. Kudin, V. N. Staroverov, T. A. Keith, R. Kobayashi, J. Normand, K. Raghavachari, A. P. Rendell, J. C. Burant, S. S. Iyengar, J. Tomasi, M. Cossi, J. M. Millam, M. Klene, C. Adamo, R. Cammi, J. W. Ochterski, R. L. Martin, K. Morokuma, O. Farkas, J. B. Foresman, D. J. Fox, *Gaussian 16 Rev. C.01*, **2016**, Wallingford, CT.

[15] F. Weigend, R. Ahlrichs, “Balanced basis sets of split valence, triple zeta valence and quadruple zeta valence quality for H to Rn: Design and assessment of accuracy“, *Phys. Chem. Chem. Phys.* **2005**, *7*, 3297-3305.

[16] V. N. Staroverov, G. E. Scuseria, J. Tao, J. P. Perdew, “Comparative assessment of a new nonempirical density functional: Molecules and hydrogen-bonded complexes“, *J. Chem. Phys.* **2003**, *119*, 12129-12137.
[17] J. Tao, J. P. Perdew, V. N. Staroverov, G. E. Scuseria, “Climbing the Density Functional Ladder: Nonempirical Meta--Generalized Gradient Approximation Designed for Molecules and Solids“, *Phys. Rev. Lett.* **2003**, *91*, 146401.
[18] R. F. Ribeiro, A. V. Marenich, C. J. Cramer, D. G. Truhlar, “Use of Solution-Phase Vibrational Frequencies in Continuum Models for the Free Energy of Solvation“, *J. Phys. Chem. B* **2011**, *115*, 14556-14562.
